# Oleaginous yeast *Rhodotorula toruloides* biomass effect on the metabolism of Arctic char (*Salvelinus alpinus*)

**DOI:** 10.3389/fmolb.2022.931946

**Published:** 2022-08-16

**Authors:** Mathilde Brunel, Viktoriia Burkina, Jana Pickova, Sabine Sampels, Ali A. Moazzami

**Affiliations:** ^1^ Department of Molecular Sciences, Faculty of Natural Resources and Agricultural Sciences, Swedish University of Agricultural Sciences, Uppsala, Sweden; ^2^ Faculty of Fisheries and Protection of Waters, South Bohemian Research Center of Aquaculture and Biodiversity of Hydrocenoses, Research Institute of Fish Culture and Hydrobiology, University of South Bohemia in České Budějovice, Vodňany, Czech Republic

**Keywords:** metabolomics, fish feed replacement, fatty acids, metabolites, gluconeogenesis, plasma, liver, oleaginous yeast

## Abstract

Sustainability issues arise when using fish oil and vegetable oils in fish feed production for aquaculture purposes. Microbial production of single cell oil is a potential alternative as a lipid ingredient in the production of fish feed. In this study, we replaced the vegetable oils with the oleaginous yeast *R. toruloides* biomass in the diet of Arctic char (*S. alpinus*) and investigated the effects on health and composition. Measurement of fish growth parameters showed a higher liver weight and hepatosomatic index in the experimental group of fish fed partly with yeast biomass compared to a control group fed a diet with vegetable oils. No significant differences in the lipid content of muscle and liver tissues were found. The fatty acid profiles in the muscle of both fish groups were similar while the experimental fish group had a higher amount of monounsaturated fatty acids in the liver. Histology of livers showed no significant difference in the number of lipid droplets. The size of hepatic lipid droplets seemed to be related to liver fat content. Quantification of metabolites in the liver revealed no differences between the fish groups while plasma metabolites involved in energy pathways such as alanine, 3-hydroxybutyrate, creatinine, serine, betaine, and choline were significantly higher in the experimental fish group.

## 1 Introduction

Over the past decades, aquaculture has steadily expanded when wild capture fisheries have stagnated. As the world’s fastest-growing food production industry, aquaculture has developed and contributes to providing animal protein, n-3 long-chain polyunsaturated fatty acids (LC-PUFAs). Aquaculture represents an essential source of income, livelihood, and nutrition for millions of people (FAO 2020). Marine products contain important high-quality lipids, proteins, and minerals required for human health (FAO, 2020). Different health agencies worldwide recommend weekly fish and fish products consumption as essential sources of LC-PUFAs, in particular eicosapentaenoic acid (EPA) and docosahexaenoic acid (DHA) whose synthesis *de novo* is not possible in humans (Bradbury, 2011; de Roos et al., 2017).

One of the limitations in the growth of aquaculture is the sustainability of all fish feed ingredients. Fish feeds are traditionally formulated with fishmeal (FM) and fish oil (FO) to fulfill protein and lipid requirements for optimal fish growth (Sprague, Betancor & Tocher, 2017). Finding appropriate alternatives to the lipid portion of fish feed is imperative as oils used for fish feed formulation are at present sourced from limited and unsustainable wild fisheries or from terrestrial plants whose current production is shared with other food products. In addition, the growing use of plant oils for fish feed might lead to increased water footprint, expansion of agricultural land, excess of nitrogen and phosphorus fertilizer leading to eutrophication, and additional use of chemical pesticides (Fry et al., 2016).

Potential alternatives and sustainable lipid sources are microbial organisms such as yeasts, bacteria, and microalgae (Sahlmann et al., 2019), with high nutritional values (Sprague, Betancor & Tocher, 2017; Gamboa-Delgado & Márquez-Reyes, 2018). Microalgae and other microorganisms are the main producer of LC-PUFAs and therefore the main provider of EPA and DHA in marine organisms (Morales-Sánchez et al., 2017; Sprague, Betancor & Tocher, 2017). An interest in cultivating heterotrophic single cell organisms (SCOs) as fish feed ingredients is growing in aquaculture (Shah et al., 2018). Heterotrophic SCOs have interesting protein and lipid productions and less demanding cultivation requirements such as lower light, and thereby volume, compared to autotrophic organisms. Moreover, heterotrophic organisms could potentially consume biomass from other biological processes as an organic carbon source for growth (Morales-Sánchez et al., 2017). SCOs have been shown to be suitable protein alternatives to FM protein in aquafeeds without detrimental effects on fish growth (Shah et al., 2018). Protein content and quality of yeasts have been previously evaluated (Sahlmann et al., 2019), specifically, proteins from baker’s yeast, showing no adverse effect on fish health, growth performance, digestibility, and nutrient retention, when incorporated in moderate amounts (Øverland et al., 2013; Øverland & Skrede, 2017; Vidakovic et al., 2020). Information on yeast as a sustainable protein source in aquaculture is available, while knowledge on yeasts’ suitability as a lipid source (oil) is limited (Øverland & Skrede, 2017; Blomqvist et al., 2018).

Oleaginous yeasts, being heterotrophic, have been considered a sustainable alternative lipid source due to their capacity to accumulate 20% and more of their dry matter mainly as triacylglycerols (TAG), their fast growth, and their ability to grow on low-value substrates such as lignocellulose hydrolysate (Abeln & Chuck, 2021). The recovery of lignocellulosic biomass to grow alternative ingredients included in fish feed production would limit the use of unsustainable resources needed for aquaculture and would repurpose by-products generated by agricultural and forestry areas (Øverland & Skrede, 2017). The costs related to the production of oleaginous yeast biomass remain more expensive than cheap plant oil such as palm oil. Nevertheless, the production costs could be limited with the use of low-cost substrates such as lignocellulosic biomass (Abeln & Chuck, 2021). With climate change, the resources and energy needed to grow plants will change drastically while the production of oleaginous yeast should not be affected (Abeln & Chuck, 2021).

Oleaginous yeast biomass has been previously included as an ingredient in fish feed with no effect on fish as demonstrated by Blomqvist et al. (2018). In that study, the oleaginous yeast *Lipomyces starkeyi* biomass was added to the diet of Arctic char (*S. alpinus*) and revealed no difference in the hepatosomatic index, condition factor, and specific growth factor.

Similar to *L. starkeyi,* the red yeast *R. toruloides* can consume lignocellulose as carbon and nitrogen sources to grow and produce lipids. The yeast *R. toruloides* was selected in our study for its higher content of polyunsaturated fatty acids compared to *L. starkeyi* strains (Brandenburg et al., 2021). In addition, *R. toruloides* yeast is capable of producing carotenoids including torulene, torularhodin, and β-carotene (Pinheiro et al., 2020; Nagaraj et al., 2022), potentially leading to a higher antioxidant activity when preserving the fish feed.

To obtain a better understanding of the effect of alternative fish feed ingredients on fish physiology, metabolomics tools can be used. Metabolomics can be defined as the non-selective study of metabolites through relative or absolute quantification (Roques et al., 2020) using nuclear resonance spectroscopy (NMR) or mass spectroscopy. The metabolites identified in an organism can be an indicator of a biological process or exposure to contaminants (xenobiotics). Fish nutrition metabolomics is a new field, as recently reviewed by Roques et al. (2020) and Lulijwa, Alfaro & Young (2022), identifying biomolecules in response to different feeds. As suggested by Roques et al. (2020), the application of metabolomics techniques in fish nutrition provides essential information on the effect of a diet on fish metabolism from a nutrient perspective and at the molecular level, which is not possible with the use of more traditional techniques such as measurement of growth parameters, digestibility, and feed conversion.

Several studies exist on the effect of diet change on fish using metabolomics when searching for more sustainable fish feed ingredients (Casu et al., 2017; Cheng et al., 2017; Wagner et al., 2019). Publications on including an oleaginous yeast biomass as a fish feed ingredient can be found (Hatlen et al., 2012; Blomqvist et al., 2018) but not from a metabolomics perspective. Therefore this study aimed to evaluate the oleaginous yeast *R. toluroides* grown on lignocellulosic biomass as a sustainable alternative ingredient in the fish feed of Arctic char and to assess the metabolic effects of this ingredient alteration in Arctic char. For that purpose, we investigated the lipid composition of the fish by measuring fatty acid profiles of muscle and liver tissues with gas chromatography, including different lipid classes of the liver. Plasma and liver metabolites compositions were studied using one proton nuclear magnetic resonance (^1^H-NMR) spectroscopy. Histological analysis was performed on livers using hematoxylin and eosin staining.

## 2 Materials and methods

**FIGURE 1:**
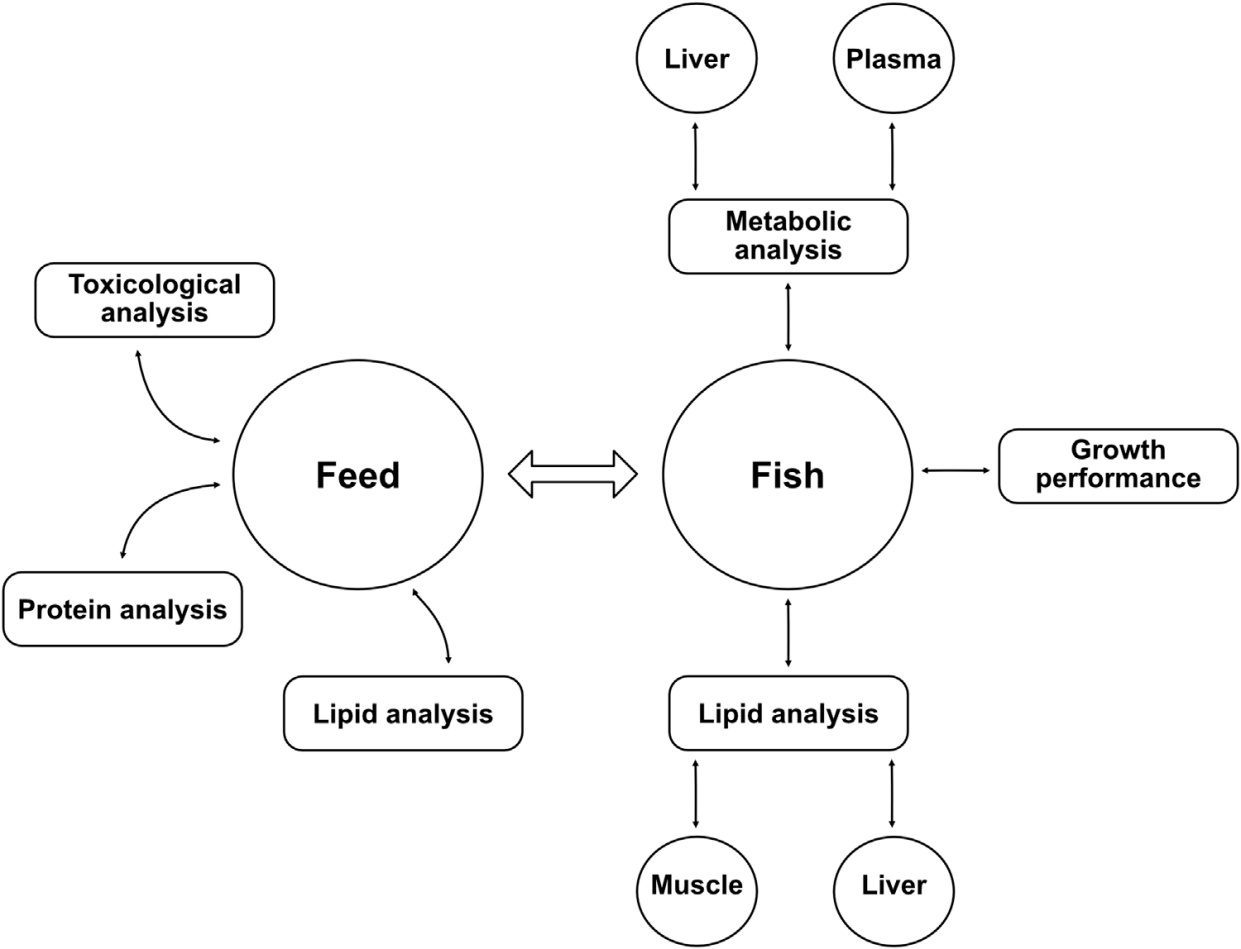
Diagram of the different analyses performed in the study. Lipid analyses were achieved with gas chromatography, metabolic analyses were conducted using nuclear magnetic resonance spectroscopy. The growth performance of fish was measured with total weight, length, and other parameters shown in Table 4. Protein analysis (crude protein content and amino acid profile) was carried out by Eurofins Food & Feed Testing Sweden AB. Toxicological analyses of the yeast biomass included measurement of organic pollutants (polycyclic aromatic hydrocarbons, polychlorinated biphenyls, and hexachlorobenzene) and heavy metals (aluminum, arsenic, cadmium, mercury, and lead) (manuscript in preparation).

A summary of the different chemical analyses conducted in this study on feed and fish can be found as a diagram in Figure 1.

### 2.1 Formulation of control and experimental feeds

Control and experimental fish feeds were formulated to contain similar protein and fat contents to meet the nutritional requirements for salmonids (Sánchez-Vázquez et al., 1999; Pettersson et al., 2009). Control feed was differentiated from the experimental feed in consisting of vegetable oils and casein while experimental feed contained no VO and no casein, and was partially made of yeast oil and proteins from the yeast biomass (Table 1).

**TABLE 1:** Composition of control and experimental yeast feeds (g kg^−1^) for fish in duplicates. “Vitamin mix” and “Mineral mix” ingredients were provided by NOFIMA (Norway) and “astaxanthin and vitamin mix” ingredients were provided by Aller Aqua A/S (Denmark).

Ingredients	Control feed	Experimental feed
Fish meal	4,950	4,950
Fish oil	1,170	1,170
Vegetable oil	540	–
Mineral mix	45	45
Vitamin mix	90	90
Astaxanthin and vitamin mix	13.5	13.5
Gelatine	45	45
Wheat meal	1.755	1.305
Casein	540	–
Ca_2_PO_4_	225	225
Yeast	-	1,413 (540 g oil)

To obtain yeast biomass, the yeast strain *R. toruloides* CBS 14 (Westerdijk Fungal Biodiversity Institute, Utrecht, Netherlands) was cultivated according to Blomqvist et al. (2018), Nagaraj et al. (2022) methods before its incorporation into the experimental feed. *R. toruloides* yeast cells were retrieved from glycerol stocks at −80°C (50% v/v) and kept on YM-agar plates at 25°C. A pre-culture of the yeast was performed before cultivation after adding a loopful of yeast cells to a 100 ml YPD-medium (Blomqvist et al., 2018). A second inoculation step was performed for 3 days under the same conditions after transferring 100 ml of yeast culture from the first inoculation to a new YPD medium. Yeast precultures were harvested by centrifugation (4,000 × g, 10 min) and washed with a saline solution (NaCl, 9 g L^−1^). *R. toruloides* was cultivated in 8 L Dolly fermentors (Belach Bioteknik, Stockholm, Sweden) at 25°C with a mix of 60% sterile filtered cellulose and 40% hemicellulose hydrolysate for 3 days (Blomqvist et al., 2018). Yeast cells were harvested by centrifugation (5,400 × g, 10 min), washed with deionized water and their cell walls were disrupted after applying a French press (Constant systems LTD, Daventry, United Kingdom). The product of the yeast cultivation was stored at −20°C until the preparation of the fish feed.

Similar composition of fatty acids was achieved between the two feeds. Fatty acids found in yeast oil were adjusted by mixing palm and rapeseed oils (1:1) in the vegetable oil mix added to control feed. All ingredients in each fish feed were added simultaneously and mixed by hand to form a homogenous paste consistency. Each fish feed was placed into a kitchen meat grinder, pressed, cut manually into pellets (2–4 mm length), and left to dry at room temperature overnight. Finally, the fish feed pellets were packed in airtight plastic bags and stored at −20°C until further use. Fatty and amino acid compositions of both feeds were analyzed (Tables 2, 3).

**TABLE 2:** Total fat content (g kg^−1^ wet weight), fatty acid composition (% of total identified FA) of the two fish feeds (duplicate analyses), and yeast biomass separately.

Fatty acids	Control feed	Experimental feed	Yeast biomass (raw)
Total fat content	19.8	18.0	29.6
14:0	4.62	4.86	
15:0	0.53	0.34	
16:0	17.6	18.7	29.4
16:1 (n-7)	4.62	4.59	
16:2 (n-4)	0.31	0.21	
17:1	0.00	0.24	
18:0	2.43	2.68	4.40
18:1 (n-9)	23.7	23.0	43.4
18:1 (n-7)	2.56	2.06	
18:2 (n-6)	6.21	5.92	16.0
18:3 (n-3)	1.86	1.47	3.50
20:1 (n-11)	1.62	1.59	
20:1 (n-9)	8.65	8.54	
18:4 (n-3)	0.32	0.34	
20:4 (n-6)	0.39	0.33	
22:1 (n-9)	11.2	11.2	
20:4 (n-3)	1.02	1.03	
20:5 (n-3)	4.95	5.12	
24:1	0.51	0.54	
22:5 (n-3)	0.65	0.50	
22:6 (n-3)	6.26	6.71	
SFA	25.2	26.6	
MUFA	52.8	51.8	
PUFA	22.0	21.6	
n-3	15.1	15.2	
n-6	6.60	6.25	
n-3/n-6	2.28	2.42	

Abbreviation: SFA, saturated fatty acids; MUFA, monounsaturated fatty acids; PUFA, polyunsaturated fatty acids.

**TABLE 3:** Amino acid composition (%) of the two fish feeds. The confidence interval of all values is below 15%. Data are presented as a proportion of total determined amino acids [% of dry matter] for both feeds. Amino acid analysis was performed by Eurofins Food & Feed Testing Sweden AB in addition to crude protein content analysis (ISO 16634-1 2008; ISO 16634-2 2016).

Amino acids	Control feed	Experimental feed
Crude protein (%)	44.8	42.1
Alanine	2.7	2.5
Arginine	2.7	2.5
Aspartic acid	4.2	3.8
Cysteine + Cystine	0.5	0.5
Glutamic acid	7.3	5.8
Glycine	2.8	2.7
Histidine	1.0	0.8
Isoleucine	1.9	1.6
Leucine	3.4	2.9
Lysine	3.5	2.8
Methionine	1.2	1.2
Phenylalanine	1.9	1.6
Proline	2.4	1.9
Serine	2.2	1.8
Threonine	1.9	1.7
Tryptophan	0.5	0.4
Tyrosine	1.6	1.3
Valine	2.2	1.9

Crude protein content and amino acid composition of both fish feeds were analyzed by Eurofins Food & Feed Testing Sweden AB (Lidköping, Sweden), following the method SS-EN ISO 13903:2005 for amino acid analysis and ISO 16634-1 2008, ISO 16634-2 2016, respectively, for crude protein content analysis.

### 2.2 Feeding trial and sample collection

Arctic char (*n* = 126, both genders, juveniles) were randomly assigned to six 1 m × 1 m water tanks (triplicate tanks per feed with 21 fish per tank) with access to a flowthrough system of freshwater (10 L min^−1^ with a water depth of 20 cm) from Lake Ansjön at Kälarne Aquaculture Center North, Sweden. Fish were acclimated for 7 days in the tanks. A natural photoperiod was kept and water temperature averaged at 7.1 ± 1.8°C. Before the feeding trial, fish were weighed, measured, and fed with a commercial feed appropriate for juvenile Arctic char. During the trial, the fish feed was distributed by band feeders 4 times a day with a feeding ratio of 2% of the actual biomass in the tanks. After 53 days of the feeding experiment, all fish were weighed and measured after a 24 h starvation period. The total body length (cm), and body and liver weights (g) were recorded for all fish. The specific growth rate (SGR) was calculated from day 19 of the trial due to technical problems occurring at the rearing station during the start of the trial.

Indicators of fish growth performance such as specific growth rate (SGR), condition factor (CF) and hepatosomatic index (HSI) were calculated as follows:

SGR = [(ln Wt−ln W0)/t] × 100
CF = Wt/T_L_^3^ × 100
HSI = (Wl/Tw) × 100

Where Wt = final weight of fish in g; W0 = initial weight of fish in g; t = time (days); T_L_ = total length in cm; Wl = weight of liver in g; Tw = total weight in g.

After the starvation period, 36 fish (6 from each tank) were anaesthetized using tricaine methanesulfonate (MS-222, 30 mg L^−1^, Sigma Chemicals Co., St. Louis, MO, United States) to overdose in a standardized way and in a well-oxygenated water, followed by a blow to the head before sampling for chemical analyses (Cheng et al., 2016b; Cheng et al., 2017) while the remaining fish were sampled for sensory analysis (manuscript in preparation).

Blood plasma was collected using venipuncture with a heparinized syringe (from 36 fish in total, with plasma quantity too low for four fish to be analyzed). Blood samples were stabilized with an aqueous solution of heparin sodium salt in Eppendorf tubes and stored at −80°C. Both liver (36 fish) and muscle tissues (dark and light fillet areas, 36 fish) were collected from the same fish according to the Cheng et al. method (2016a; 2016b) and stored at −80°C until analyses. During the chemical analysis, 32 plasma samples and 12 liver samples (two from each tank) were analyzed for the quantification of metabolites. From a total of 36 muscle samples, 12 samples (two from each tank) were analyzed for lipid content and fatty acid profiles with technical replicates.

The survival rate of the fish during the feeding trial was at its maximum with no death recorded. The experiment was approved by the Ethical Committee for Animal Experiments (Umeå, Sweden) and carried out in compliance with the European legislation (i.e., Directive 2010/63/EU).

### 2.3 Lipid extraction and fatty acid analyses

#### 2.3.1 Lipid extraction

Total fat content and FA composition of muscle and liver tissues as well as of the two feeds were determined using a lipid extraction method with gas-liquid chromatography. Total lipids were extracted from fillets (*n* = 12), liver (*n* = 36), and the fish feed with approx. 2 g suspended in 8 ml H_2_O, in technical duplicates (Hara & Radin, 1978; Mráz & Pickova, 2009). Briefly, 1 g of sample was homogenized in the hexane-isopropanol mix (HIP; 3:2, *v:v*) three times for 30 s with an Ultra-Turrax (Janke and Kunkel, IKA Werke, Germany). Lipids were separated from non-lipids after adding 6 ml of Na_2_SO_4_ solution (6.67%, *w:v*) and mixing with a vortex. The samples were left for separation at 4°C for 15 min and the transferred lipid phase was dried under nitrogen gas. The total lipid content was quantified gravimetrically and lipids were stored in hexane −80°C until further analysis.

#### 2.3.2 Lipid classes

In addition to total fat content and FA composition analyses, total extracted lipids (TL) from fish liver were separated into phospholipids (PL) and triacylglycerols (TAG) classes using thin layer chromatography (TLC) as described earlier by Pickova et al. (1997) and Mráz & Pickova (2009). TLC plates (20 cm × 20 cm; pre-coated Silica gel 60; 0.20-mm thickness from Merck, Darmstadt, Germany) were selected as stationary phase and solvents (hexane, diethylether, and acetic acid; 85:15:1, v/v/v) were added to TLC chamber. PL and TAG sample areas were identified by comparison with a reference standard separately revealed in iodine. Areas of interest were scraped off the TLC plates. The PL class was extracted successively with chloroform:methanol (2:1, 2 ml, v:v); chloroform:methanol (1:1, 3 ml, v:v) and chloroform (2 ml), while TAG was extracted three times in succession with chloroform (2 ml). Samples were evaporated under nitrogen gas, recovered in 0.5 ml hexane, and stored at −20°C until further analysis.

#### 2.3.3 Methylation and gas chromatography

From the total extracted lipids of fish feed, fish muscles, and liver, 2 mg of each sample was taken for identification of FA composition after a methylation step using boron trifluoride (BF3, 14%) and methanol reagents according to the method described by Appelqvist (1968). Briefly, methylation was performed by adding NaOH (2 ml, 0.01 M) in dry methanol in each sample followed by an incubation time of 10 min at 60°C. A similar next step was completed with each sample by adding BF3 (3 ml, 14%) with another incubation time of 10 min at 60°C. After cooling, NaCl (2 ml, 20%) and hexane (2 ml) were added simultaneously to each sample and left to separate. The upper phase containing hexane and FA methyl esters (FAME) was transferred to a new tube and evaporated under nitrogen gas. The same methylation method was applied to the different liver lipid classes (PL and TAG) using the entire sample. FAME generated after methylation were stored in hexane at −80°C until analyses.

The FAME were analyzed by GC with a CP 3800 instrument (Varian AB, Stockholm, Sweden) equipped with a flame ionization detector, and a split-mode injector, and separated on a 50 m fused silica capillary column BPX 70 (SGE, Austin, Tex) with 0.22 mm i.d. × 0.25 μm film thickness. The injector and detector temperatures were 230°C and 250°C, respectively. Helium at a flow rate of 0.8 ml min^−1^ was the carrier gas while nitrogen was the make-up gas. Identification of FA peaks was achieved by comparing retention times of each FA peak with those of the standard mixture GLC-68A (Nu-check Prep, Elysian, United States). Peak areas were integrated using the Galaxie chromatography data system software version 1.9 (Varian AB, Stockholm, Sweden).

### 2.4 Liver and plasma metabolomics using ^1^H-NMR spectroscopy

Fish liver tissue was extracted according to previous studies (Moazzami, Andersson & Kamal-Eldin, 2011; Wagner et al., 2019) with minor modifications. Briefly, the frozen liver tissue sample (100 mg) was homogenized with an Ultra-Turrax (Janke and Kunkel, IKA Werke, Germany) in an ice-cold methanol-chloroform mix (2:1, *v:v*, 3 ml) for 1 min and sonicated for 30 min. After adding 1 ml of ice-cold chloroform and 1 ml of ice-cold water, each sample was vortexed for 1 min before centrifugation at 1,800 g × for 35 min at 4°C. The aqueous supernatant (polar phase) was collected, dried using a speedvac (Savan, SVC 100H, Techtum Instrument AB, Umeå, Sweden), and redissolved in 520 µl sodium phosphate buffer (0.135 M, pH 7.0). Reconstituted samples were filtered using pre-washed Nanosep centrifugal filters with a 3-kDa cutoff (Pall Life Science, Port Washington, NY) by centrifugation at 12,000 × g at 4°C for at least 2 h. For each sample, fish liver filtrate (350 µl), sodium phosphate buffer (170 μl, 0.135 M, pH 7), deuterium oxide (D_2_O, 50 µl), and the internal standard sodium-3-(trimethylsilyl)-2,2,3,3-tetradeuteriopropionate solution (TSP-d4, 30 μl, 5.8 mmol/L, Cambridge Isotope Laboratories, Andover, MA, United States) were mixed and added to a 5-mm outer diameter NMR tube (Bruker Spectrospin Ltd., BioSpin, Karlsruhe, Germany) before analysis.

Plasma samples were filtered following a similar method to Röhnisch et al. (2018), using pre-washed Nanosep centrifugal filters with a 3-kDa cutoff (Pall Life Science, Port Washington, NY). Samples were added to filters and centrifuged at 10,000 × g, 4°C for at least 1 h 30 min. For quantification of metabolites, a mix containing filtrate (40 µl), sodium phosphate buffer (50 μl, 0.4 M, pH 7), D_2_O (15 µl), Millipore water (55 µl), and TSP-d_4_ (10 μl, 5.8 mmol/L, Cambridge Isotope Laboratories, Andover, MA, United States) was prepared for each sample and added to a 3-mm outer diameter NMR tube.

Both plasma and liver analyses were performed on the same Bruker spectrometer instrument operating at 600 MHz equipped with a cryogenically cooled probe and autosampler. ^1^H-NMR spectra were acquired using a zgesgp pulse sequence (Bruker Spectrospin Ltd.) at 25°C, over a spectral width of 17,942.58 Hz (acquisition time: 1.83 s, relaxation delay 4 s) at 65,536 data points with 512 scans for plasma samples and 128 scans for liver samples. Data were processed using Bruker Topspin 4.1.1 software and Fourier-transformed after multiplication by line broadening of 0.3 Hz. NMR spectra had their baseline adjusted manually and were calibrated using TSP-d_4_ at 0.0 ppm. ChenomX NMR suite version 7.1 profiler (ChenomX Inc., Edmonton, AB, Canada), the Human Metabolome database as well as previous literature (Cheng et al., 2017) were used to identify and quantify metabolites in plasma and liver samples.

### 2.5 Histological preparation of fish livers

Fish livers (*n* = 8) were prepared for histological analysis using hematoxylin (Mayers HTX, HistoLab) and eosin (Eosin Y 0.2%, HistoLab) staining.

The samples kept at −80°C were first thawed and fixed in neutral buffered formaldehyde solution (10%) for 24 h. The livers were sliced (size 4 mm) and placed in cassettes for embedding. The sliced livers were prepared for dehydration and paraffin infiltration using a tissue processing machine (Thermo Scientific Excelsior AS) for 13–14 h. The samples were embedded in paraffin blocks, sectioned with a microtome (Thermo Scientific HM 355S, section size = 4 µm), and placed on glass slides. The slides were dried overnight at 37°C and later incubated at 60°C for 40 min. Several rehydration steps were performed on the slides with xylene solution and with two concentrations of ethanol (95 % and 70%). The samples were stained with Mayer’s hematoxylin (5 min) and with eosin (30–60 s). Dehydration steps were performed with ethanol (95%) followed by cleaning steps with xylene solution before mounting the slides with coverslips.

Histological slides were analyzed using a light microscope (Nikon Eclipse Ni) mounted with a camera (Nikon Digital sight DS-Fi2). Images were captured with NIS-Elements Basic Research (Nikon Imaging Software).

Lipid droplets were counted at the same area of the same size of an image (1.44 cm × 1.44 cm) using the grid lines option in ImageJ with 60,000 pixels^2 in area per point (Focus 33.3%).

### 2.6 Statistics

Differences in growth parameters were estimated using a PROC MIXED function in SAS software (version 9.4, SAS Institute Inc, Cary, NC, United States), taking into consideration the different fish tanks as a random factor. The same statistical method was applied to total fat content, FA in lipid classes, and FA in total lipids from liver and muscle samples.

Multivariate data analysis was performed on liver and plasma metabolites using the SIMCA software (version 17; Umetrics, Suite of Data Analytics Solutions, Sartorius). Data were UV-scaled and principal component analysis (PCA-X) scores were plotted to detect strong outliers (Eriksson et al., 2013) using Hotelling’s T2 (CI 95-99%). No strong or moderate outliers were observed in a dataset of liver samples. Two outliers (strong or moderate) were identified in the dataset of plasma samples: fish samples 1 and 6. Interpretation of data was performed with and without the outliers in the plasma dataset.

After a search for outliers, liver and plasma data were classified using control/experimental feeds as a discriminative variable. The data classification to assess the difference between the two groups of fed fish was performed using an orthogonal partial least-squares discriminant analysis (OPLS-DA) model.

The reliability of the OPLS-DA models was verified with analysis of variance testing of cross-validated predictive residuals (CV-ANOVA), determined on an ANOVA assessment of the cross-validatory predictive residuals of some types of the models such as PLS and OPLS models. CV-ANOVA is based on significance testing using an F-distribution by comparing two models fitted to the same dataset by their fitted residuals (Eriksson et al., 2008). In addition, to confirm the validity and predictability of the different multivariate models, R^2^X (proportion of variation modeled in the component, using the X-model) and Q^2^ (proportion of variation predicted by the model X in that component according to predicted residuals) parameters were reported.

Discriminative metabolites were classified in OPLS-DA models based on their variable influence on projection (VIP) value. Metabolites with VIP values > 1 and with the corresponding jackknife-based 95% confidence intervals (CIs) above or including zero were considered discriminative. All metabolites were evaluated by the PROC MIXED function in SAS 9.4 with tank factor as a random factor but only metabolites meeting criteria in SIMCA were considered to have a significant difference between the two fish groups in this study.

Metabolites appearing significantly different between the fish groups in SIMCA were checked for normality with the PROC MIXED function in SAS 9.4 including tank factor. Metabolites not normally distributed were tested for significance in SAS 9.4 using NPAR1WAY with Wilcoxon score.

Regarding histology data (*n* = 8), the statistical difference in the numbers of lipid droplets between the two fish groups was evaluated in SAS 9.4 with the PROC MIXED function, taking into consideration the different fish tanks as a random factor. In addition, a multivariate analysis with the PCA-X score plot (SIMCA software, version 17; Umetrics, Umeå, Sweden) was added to the interpretation of histological data. Fat content (%) and number of lipid droplets were included in the PCA model as variables with UV-scaled data.

## 3 Results

### 3.1 Fish performance

Fish showed a similar growth during the feeding trial with no differences observed in the final weight, final length, and specific growth factor (Table 4). Nevertheless, fish fed with the experimental feed depicted a significantly higher liver weight and higher hepatosomatic index.

**TABLE 4:** Growth parameters for fish fed with control feed or experimental yeast-based feed. Data are presented as the mean ± standard deviation. Statistical significance was set at *p*-value < 0.05.

Growth parameters	Control group	Experimental group	*p*-value
Weight (g), day 19	209 ± 65.4	200 ± 62.9	0.7360
(n = 12 per group)			
Final length (cm)	29.4 ± 2.20	29.2 ± 2.54	0.7417
(n = 48 per group)			
Final weight (g)	314 ± 99.1	303 ± 94.3	0.6108
(n = 48 per group)			
**Liver weight (g)**	**3.75 ± 2.07**	**4.61 ± 1.91**	**0.0069**
(n = 48 per group)			
SGR (%)	0.68 ± 0.59	0.70 ± 0.56	0.8455
(53 days)			
CF (%)	1.20 ± 0.14	1.19 ± 0.22	0.9078
(n = 48 per group)			
**HSI (%)**	**1.14 ± 0.27**	**1.51 ± 0.34**	**<0.0001**
(n = 48 per group)			

Significant results are shown in bold letters. SGR, specific growth rate; CF, condition factor; HSI, hepatosomatic index.

### 3.2 Fatty acid profiles in muscle and liver tissues

Total fat content and fatty acid profiles were of the same levels in muscle tissue of the fish between control and experimental feeds (Table 5). In the same way, the total fat content from the fish liver tissue (Table 6) was similar between the two groups of fish.

**TABLE 5:** Total fat content (%) and fatty acid profile in the fillet (dark and light muscle; % of total identified FA) from Arctic char fed with control feed and experimental yeast feed. Data are presented as mean ± standard deviation, (*n* = 12 in total, 3 technical replicates). Statistical significance was set at *p*-value < 0.05.

Fatty acid composition (%)	Control group (*n* = 6)	Experimental group (*n* = 6)	*p*-value
Total fat content (%)	5.95 ± 2.06	6.46 ± 1.37	0.6335
C14:0	4.04 ± 0.13	4.07 ± 0.16	0.7665
C15:0	0.33 ± 0.16	0.29 ± 0.10	0.7185
C16:0	15.4 ± 0.60	15.2 ± 0.33	0.5582
C16:1 (n-9)	0.41 ± 0.12	0.29 ± 0.07	0.3931
C16:1 (n-7)	5.28 ± 0.44	5.81 ± 0.37	0.0663
C18:0	2.18 ± 0.13	2.13 ± 0.10	0.5287
C18:1 (n-9)	26.8 ± 1.39	28.0 ± 0.98	0.1577
C18:1 (n-7)	2.44 ± 1.19	2.33 ± 0.97	0.8783
C18:2 (n-6)	6.80 ± 0.30	6.73 ± 0.17	0.6589
C18:3 (n-3)	1.96 ± 0.21	1.79 ± 0.27	0.3988
C20:1 (n-9)	6.98 ± 0.30	7.01 ± 0.29	0.8422
C20:2 (n-6)	0.54 ± 0.09	0.43 ± 0.05	0.2310
C20:4 (n-6)	0.51 ± 0.03	0.40 ± 0.11	0.2120
C20:5 (n-3)	4.11 ± 0.31	3.94 ± 0.21	0.3049
C24:1	0.58 ± 0.11	0.51 ± 0.14	0.3955
C22:5 (n-3)	1.05 ± 0.13	1.04 ± 0.15	0.9454
C22:6 (n-3)	11.3 ± 0.96	11.0 ± 1.06	0.6134
SFA	22.0 ± 0.82	21.7 ± 0.31	0.4415
MUFA	43.6 ± 1.63	44.7 ± 1.44	0.2706
PUFA	33.2 ± 0.96	32.3 ± 1.17	0.1980
n-3	25.5 ± 1.01	25.1 ± 1.36	0.5265
n-6	7.65 ± 0.35	7.22 ± 0.38	0.0893
n-3/n-6	3.35 ± 0.24	3.49 ± 0.34	0.4579

Abbreviation: SFA, saturated fatty acids; MUFA, monounsaturated fatty acids; PUFA, polyunsaturated fatty acids.

**TABLE 6:** Total fat content of liver and TL, PL, TAG fatty acid composition (% of total identified FA) of the control fed fish and experimental (yeast) fed fish. Data are presented as mean ± standard deviation, (*n* = 36 in total with 18 from each feed group, 3 technical replicates). Statistical significance was set at *p*-value < 0.05.

	TL			TAG			PL		
	Control group	Experimental group	*p*-value	Control group	Experimental group	*p*-value	Control group<	Experimental group	*p*-value
	Mean ± SD	Mean ± SD		Mean ± SD	Mean ± SD		Mean ± SD	Mean ± SD	
Fat content (%)	14.5 ± 6.60	17.2 ± 4.68	0.1785						
C14:0	2.60 ± 0.37	2.71 ± 0.27	0.5556	2.88 ± 0.23	2.96 ± 0.23	0.3294	n.d	n.d	—
C16:0	12.5 ± 1.23	12.3 ± 0.85	0.7345	10.3 ± 1.25	10.7 ± 2.77	0.5742	17.9 ± 1.21	17.3 ± 1.26	0.1981
C16:1 (n-9)	0.50 ± 0.05	0.57 ± 0.06	0.0020	0.52 ± 0.06	0.59 ± 0.07	0.0047	n.d	n.d	—
C16:1 (n-7)	8.17 ± 1.93	9.44 ± 0.98	0.0557	9.62 ± 1.24	10.4 ± 1.04	0.0669	0.93 ± 0.22	1.16 ± 0.31	0.1159
C18:0	2.15 ± 0.33	2.39 ± 0.48	0.2981	1.84 ± 0.32	2.26 ± 0.58	0.1079	5.75 ± 1.03	6.49 ± 0.91	0.1717
C18:1 (n-9)	34.1 ± 4.82	38.1 ± 2.65	0.0047	39.7 ± 1.26	41.5 ± 2.25	0.0180	13.6 ± 0.80	13.9 ± 0.85	0.2401
C18:1 (n-7)	3.93 ± 0.42	3.69 ± 0.22	0.1434	4.46 ± 0.24	3.95 ± 0.26	0.0002	2.61 ± 0.30	2.65 ± 0.35	0.7859
C18:1 (n-5)	n.d	n.d	—	0.34 ± 0.11	0.29 ± 0.02	0.5768	n.d	n.d	—
C18:2 (n-6)	3.89 ± 0.45	3.41 ± 0.47	0.0090	4.53 ± 0.92	3.67 ± 0.58	0.0013	2.25 ± 0.25	2.12 ± 0.22	0.1786
C18:3 (n-6)	n.d	n.d	—	0.35 ± 0.05	0.37 ± 0.07	0.7049	n.d	n.d	—
C18:3 (n-3)	0.79 ± 0.13	0.57 ± 0.15	<0.0001	0.94 ± 0.23	0.61 ± 0.17	<0.0001	n.d	n.d	—
C20:1 (n-9)	7.18 ± 0.85	7.56 ± 0.51	0.1234	8.65 ± 0.49	8.38 ± 0.51	0.1123	3.33 ± 0.71	3.49 ± 0.83	0.7114
C20:2 (n-6)	0.62 ± 0.07	0.59 ± 0.06	0.2492	0.68 ± 0.08	0.62 ± 0.07	0.0180	n.d	n.d	—
C20:3 (n-6)	0.63 ± 0.40	0.50 ± 0.07	0.4002	0.41 ± 0.05	0.49 ± 0.09	0.3054	n.d	n.d	—
C20:4 (n-6)	1.02 ± 0.51	0.70 ± 0.20	0.0226	n.d	n.d	—	4.51 ± 0.38	4.46 ± 0.50	0.7732
C22:1 (n-9)	0.69 ± 0.09	0.71 ± 0.05	0.6317	0.87 ± 0.07	0.85 ± 0.10	0.6650	n.d	n.d	—
C20:5 (n-3)	4.57 ± 1.26	3.54 ± 0.58	0.0037	3.75 ± 0.84	3.08 ± 0.55	0.0099	7.24 ± 0.79	7.02 ± 0.65	0.4693
C24:1	0.56 ± 0.10	0.47 ± 0.09	0.0925	0.64 ± 0.08	0.53 ± 0.09	0.0013	n.d	n.d	—
C22:5 (n-3)	1.42 ± 0.36	1.38 ± 0.19	0.8117	1.58 ± 0.27	1.36 ± 0.23	0.0630	1.18 ± 0.12	1.32 ± 0.12	0.0118
C22:6 (n-3)	15.6 ± 5.47	11.9 ± 1.78	0.0112	8.91 ± 1.47	8.10 ± 0.94	0.0582	40.6 ± 2.08	41.1 ± 1.63	0.6127
SFA	17.2 ± 1.35	17.4 ± 1.13	0.6395	15.1 ± 1.53	16.0 ± 2.79	0.5411	23.9 ± 1.89	23.8 ± 1.55	0.9334
MUFA	54.8 ± 7.86	60.4 ± 3.10	0.0087	64.4 ± 2.05	66.2 ± 2.69	0.0266	20.1 ± 1.80	20.4 ± 1.42	0.5739
PUFA	27.9 ± 7.24	22.2 ± 3.06	0.0043	20.6 ± 3.17	17.8 ± 2.45	0.0064	55.7 ± 2.56	55.7 ± 1.49	0.9827
n-3	22.3 ± 6.70	17.3 ± 2.43	0.0053	15.2 ± 2.46	13.0 ± 1.70	0.0049	48.9 ± 2.16	49.1 ± 1.33	0.8216
n-6	5.58 ± 0.79	4.90 ± 0.81	0.0157	5.40 ± 1.02	4.77 ± 0.93	0.1397	6.87 ± 0.56	6.62 ± 0.49	0.1845
n-3/n-6	3.97 ± 0.86	3.56 ± 0.40	0.0789	2.85 ± 0.42	2.78 ± 0.41	0.7099	7.14 ± 0.44	7.46 ± 0.60	0.0808

TL, total lipids; PL, phospholipids; TAG, triacylglycerols; SFA, saturated fatty acids; MUFA, monounsaturated fatty acids; PUFA, polyunsaturated fatty acids; n.d, non-detected.

Differences were observed in the percentages of fatty acids in total lipids (TL) and triacylglycerols (TAG) of the liver between the differently fed fish (Table 6). In TL and TAG, the liver of fish fed with control feed showed higher percentages of PUFA and n-3 (in particular arachidonic, linoleic, and alpha-linolenic acids) whereas the liver of fish fed with experimental feed contained more MUFA with the exception of nervonic acid higher in TAG class of control group. Levels of EPA were significantly higher in TL and TAG lipid classes in the control group of fish while docosapentaenoic acid (DPA) was exclusively higher in PL class of the experimental group. Levels of DHA were solely higher in TL class in the control group of fish.

Levels of SFA in the liver remained similar between the two fish groups with the same level of palmitic acid in all lipid classes of the liver for both control and experimental fed fish groups (Table 6).

Fatty acid percentages in phospholipids (PL) of the liver were generally similar between the two groups of fish with the exception of DPA significantly higher in liver of the experimental fish group and ratio n-3/n-6 slightly higher in the same fish group (Table 6).

### 3.3 Metabolomic profiles of blood plasma and liver tissue from fish

Different metabolites were detected in fish liver and plasma samples from different classes: amino acids; lipids and lipid-like molecules; nucleosides, nucleotides and analogs; organic acids, and more to obtain a broad representation of the different activities occurring in several fish metabolic pathways.

In fish liver tissue, 48 metabolites were quantified and no strong outliers were detected according to the PCA-X score plotted (Supplementary Figure S1). The fitted PCA model was explained by two principal components (PCs), with the following model parameters R^2^X = 58.3%, R^2^X2 = 11.7%, Q^2^ = 44.3%. The separation was not clear with the PCA score plot but became clearer with the OLPS-DA score plot (all data) with one predictive (P1) and two orthogonal (O2) components, including the model parameters: R^2^X = 12.6%, Q^2^X = 24.7%, R^2^Y = 100%. CV-ANOVA value (*p*-value = 0.84) indicated that the OPLS-DA model could not be considered reliable for predictions. After classification of metabolites in a list according to VIP and CI values and verification of normality and significance using SIMCA software, no metabolite appeared significantly different in concentrations between the fish fed with control feed and the fish fed with experimental feed as seen in Table 7 (Supplementary Table S1).

**TABLE 7:** Metabolites in plasma in μM L^−1^ (*n* = 36 in total with 4 missing values) and liver in µM g^−1^ (*n* = 12), (total plasma metabolites = 57 and total liver metabolites = 48). Data are presented as median (Q1-Q3) with concentrations and *p*-value obtained from PROC MIXED function in SAS 9.4 with tank factor. VIP values were retrieved from SIMCA. Statistical significance was set at *p*-value < 0.05. Plasma results are presented without outliers fish 1 and fish 6.

Metabolites	Control group	Experimental group	VIP (95% CI)	*p*-value
*Plasma* µM L^−1^	n = 16	n = 14
Propylene glycol	6.38 (5.10–7.44)	29.54 (25.93–32.30)	1.75	<0.0001
Alanine	480.25 (456.03–551.44)	758.41 (619.23–898.45)	1.38	0.0002
3-Hydroxybutyrate	5.53 (3.83–6.38)	11.69 (9.78–14.88)	1.28	0.0004
N,N-Dimethylglycine	1.70 (1.70–2.13)	2.55 (1.70–3.40)	1.01	0.0413
Creatinine	18.06 (15.94–21.25)	24.23 (22.53–25.50)	0.91	0.0005
Histidine	75.44 (60.14–87.13)	56.53 (51.00–65.45)	0.58	0.0765
Choline	46.11 (35.70–50.79)	58.23 (51.85–62.90)	0.44	0.0069
Taurine	1420.56 (1234.63–1814.54)	1243.76 (1115.20–1341.30)	0.41	0.0630
Lactate	5371.15 (4376.01–6001.43)	6527.79 (5001.40–7247.10)	0.17	0.0413
Inosine	101.58 (82.24–128.14)	112.20 (103.28–233.33)	0.14	0.1157
Betaine	30.60 (19.98–47.60)	47.39 (41.65–66.30)	0.13	0.0138
Tyrosine	93.93 (77.56–107.95)	76.93 (64.60–80.33)	0.09	0.0089
Serine	167.88 (151.09–198.90)	226.53 (185.30–240.98)	0.04	0.0132
Phenylalanine	97.96 (89.04–109.86)	91.38 (87.98–99.88)	0.04	0.1202
Glycine	440.51 (369.54–511.70)	544.64 (489.18–643.45)	0.03	0.0354
*Liver* µM g^−1^	n = 6	n = 6
Tryptophan	0.288 (0.222–0.505)	0.151 (0.096–0.179)	0.47	0.0669
S-Adenosylhomocysteine	0.043 (0.035–0.051)	0.031 (0.026–0.032)	0.35	0.2609
Aspartate	1.149 (0.525–1.231)	0.435 (0.284–0.860)	0.33	0.1653
Glutamate	2.674 (1.757–4.787)	2.112 (1.269–2.393)	0.32	0.4382
Glutamine	1.626 (1.153–2.351)	1.383 (0.891–1.592)	0.28	0.4426
Fumarate	0.147 (0.080–0.201)	0.067 (0.053–0.086)	0.19	0.2361
Histamine	0.708 (0.340–0.813)	0.544 (0.287–0.571)	0.07	0.3969

**FIGURE 2:**
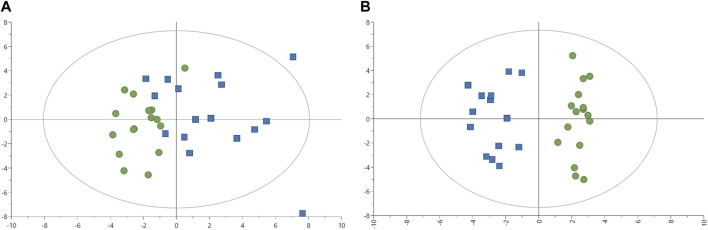
Image **(A)** Score plot of principal component analysis (PCA) model of ^1^H-NMR data plasma (*n* = 32). Green circles: fish-fed control feed. Blue squares: fish fed with experimental feed. PCA score plot (all plasma data, with 2 PCs) with parameters: R^2^X = 16.4%, R^2^X2 = 13.4%, Q^2^X = 1.74%. Image **(B)** Score plot of an orthogonal partial least squares-discriminant analysis (OPLS-DA) with one predictive (P1) and two orthogonal (O2) components) model of ^1^H-NMR data plasma. Green circles: fish-fed control feed. Blue squares: fish fed with experimental feed. OPLS-DA score plot (plasma samples without outliers) with R^2^X = 13.1%, Q^2^X = 79.0%, R^2^Y = 100%, CV-ANOVA = 9.02 × 10^−07^.

The effect of the two feeds on fish was evaluated in fish plasma with 57 metabolites quantified. Two outliers were identified after plotting the PCA-X score with plasma samples from fish one and six removed for further analyses (Figure 2A). The PCA model without two outliers was estimated using two principal components with the model parameters: R^2^X = 16.0%, R^2^X2 = 13.8%, Q^2^X = 0.66%. Similar to liver analysis, no clear pattern appeared in metabolites from plasma samples with the PCA-X score plot. A better distinction was made after plotting the OPLS-DA score without two outliers (Figure 2B), with one predictive and two orthogonal components with the following model parameters: R^2^X = 13.1%, Q^2^X = 79.0%, R^2^Y = 100%. CV-ANOVA showed better predictability of the plasma OPLS-DA model than of the liver OPLS-DA model with *p*-value = 9.02 × 10^−07^. Several metabolites were identified as discriminative variables between the two groups of fish with propylene glycol, alanine, and 3-hydroxybutyrate indicating the strongest difference between the two fish groups as seen in Table 7 (Supplementary Table S2). Most plasma metabolites significantly different between the two fish groups were in higher concentrations in fish fed with experimental feed with the exception of tyrosine, and higher in control-fed fish.

### 3.4 Histology results

**FIGURE 3:**
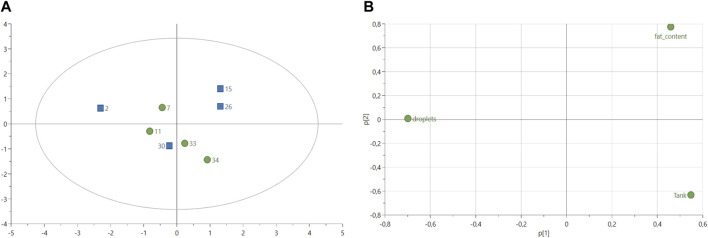
**(A)** Score plot of principal component analysis (PCA) model of lipid droplets calculated from histological data (*n* = 8 in total, four samples per treatment). Variable fish tanks and fat content (%) were included in the analysis. Green circles: fish-fed control feed. Blue squares: fish fed with experimental feed. PCA score plot (all data, with 2 PCs) with parameters: R^2^X = 50.4%, R^2^X2 = 32.6%, Q^2^X = −36.6%. **(B)** Loadings plot from PCA model shown in **(A)**.

The histology analysis of fish livers (*n* = 8 in total, 4 in each group) from both control and experimental groups revealed no difference in the number of lipid droplets for the same size area of 1.44 cm^2^ (Table 8) with on average 4.69 droplets in livers of fish fed with experimental diet and 4.45 droplets in livers of fish fed with control diet (Supplementary Table S3). The PCA score plot of the variables “number of lipid droplets,” “fat content (%)” and fish “tank” showed similarities between the experimental fish number 30 and all control fish (Figure 3A). Experimental fish numbers 15 and 26 were differentiated from other samples with a higher fat content according to the loadings (Figure 3B). Fish number two from the experimental group showed the highest number of lipid droplets compared to other fish (Figure 3B).

**TABLE 8:** Histological analyses of lipid droplets count in liver tissue (*n* = 8 in total with 4 fish per feed group). Data are presented as mean ± standard deviation. Statistical significance was set at *p*-value < 0.05.

Fish group	Droplets (per 1.44 cm^2^)	*p*-value
Control	4.45 ± 0.40	0.7032
Experimental	4.69 ± 0.85	

**FIGURE 4:**
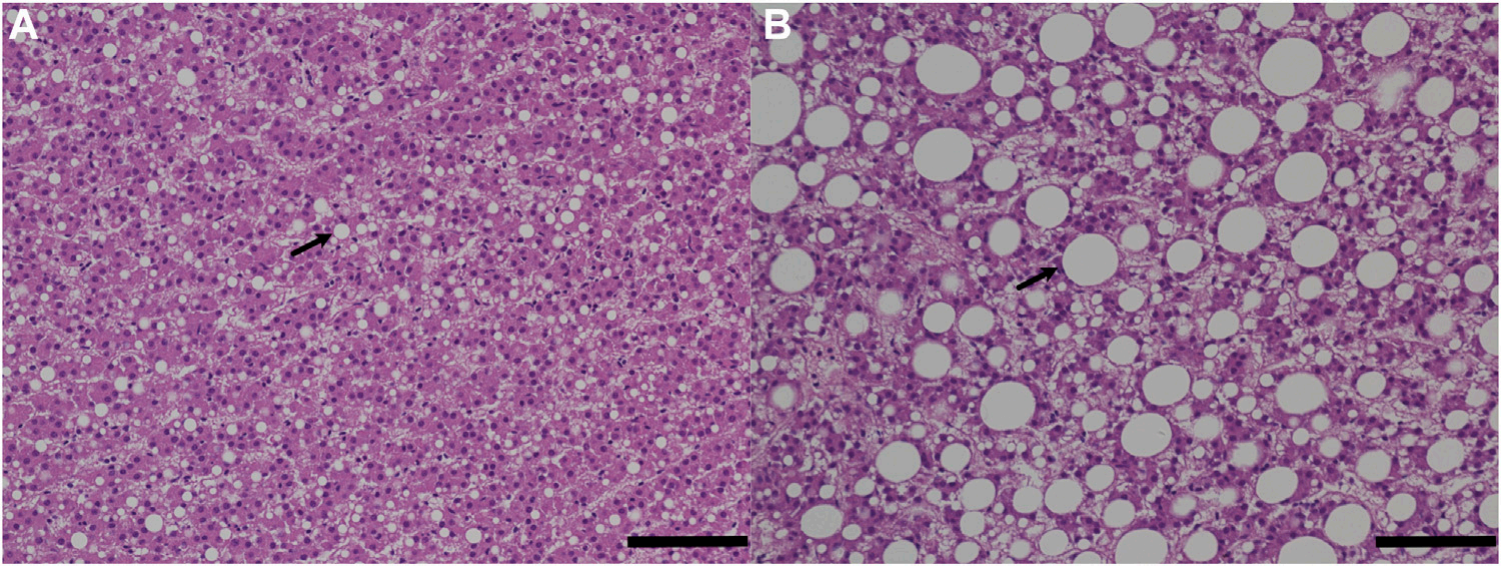
Histological analysis of fish liver tissues. The bar scale represents 100 µm. The image on the left **(A)** is control fish, and the image on the right **(B)** is experimental fish with signs of hepatic steatosis. Arrows point out lipid droplets.

The histological phenotypic results were consistent with the measured lipid content in the fish liver with two fish fed with experimental feed (fish number 15 and 26) showing the highest liver fat content (>20%) and the largest lipid droplets compared to any other fish from the control group (Figure 4; Supplementary Table S3).

## 4 Discussion

The search for alternatives to animal and vegetable lipids leads to the expansion of SCOs knowledge and broader utilization of microbial oils. The industrial application of microbial oils as lipid sources shows advantages such as the potential use for foods and feeds, biofuel production, the recovery of carbon, nitrogen, minerals, and other nutrients from under-utilized industrial by-products (Øverland & Skrede, 2017; Abeln & Chuck, 2021). Microbial production of oil remains expensive for common use as biodiesel. Nevertheless, optimizations through higher lipid yield, improved fermentation processes and the use of low cost substrates could reduce the costs (Abeln & Chuck, 2021). Microbial oil could bring substantial benefits to the environment with limited reliability on the climate as the fatty acid profile of some SCOs remains similar to the one from terrestrial plant oils (Abeln & Chuck, 2021). Oleaginous yeasts from the *Rhodotorula* genus, rich in palmitic acid, could replace palm oil in animal feed and bring other advantages as they grow rapidly and are an important source of carotenoids with antioxidant properties (Nagaraj, et al., 2022).

In the present study, *R. toruloides* was selected as a vegetable oil replacement in the diet of Arctic char, and feeds were designed to have similar fat content and fatty acid composition. The total fat content and fatty acids profile in the muscle of both the control fish fed with a vegetable oil mix and the experimental groups of fish fed partly with yeast biomass were similar, indicating that lipids from the biomass were available for uptake and similar metabolism of the feeds. These results are in agreement with Blomqvist et al. (2018) study using *L. starkeyi*. Former studies have demonstrated a mirroring effect of the fish feed on Arctic char muscle lipid composition (Pettersson, Pickova & Brännäs., 2010; Dupont-Cyr et al., 2022). Therefore, the evaluation of fatty acid profile and lipid content in fish muscle was required when evaluating fish growth performance as a modification of the fish feed may change its lipid composition, possibly leading to effects on the fish performance such as swimming performance and total lipid content (Pettersson, Pickova & Brännäs., 2010) on growth factors (Wagner et al., 2014), or a change on fatty acid composition of fillets for human consumption (Teoh & Ng., 2016).

In the same way as muscle tissue, liver total lipid content was not significantly different between the two groups of fish, although it was slightly higher in the experimental group. As the main organ for lipid and carbohydrate storage in most fish species, the liver is commonly used as a site of biomarkers in the dysregulation of fatty acid metabolism (Monge-Ortiz et al., 2018; Roques et al., 2020). The fatty acid profile of the different lipid classes of the liver revealed differences between fish groups with higher PUFA content in the control group for TL and TAG, higher MUFA amounts in the experimental group for TL and TAG, and no difference in SFA contents between the fish groups for any lipid class. In the present study, DHA as essential FA in the PL for both fish groups with 40%, was not affected by the different feeds, indicating its importance and thereby retention. Levels of C18:2 (n-6) and n-6 were significantly higher in liver TL and TAG of fish fed with control feed. Higher levels of C18:2 (n-6) have been observed before in fish feed based on plant oils, in particular, soybean oil and rapeseed oil compared to fish feed based on fish oil (Thomassen & Røsjø, 1989; Kalogeropoulos, Alexis & Henderson, 1992; Willora et al., 2021). Higher levels of C18:2 (n-6) in fish feed have shown to be mirrored in the fatty acid composition of the fish liver as seen in Kalogeropoulos, Alexis & Henderson (1992) with a proportional increase of C18:2 (n-6) in liver phospholipids of fish fed with an increase of soybean oil quantity in the fish feed. In the current study, significantly higher levels of C18:2 (n-6) observed in the liver of the fish control group were not explained by the fatty acid profile of the feeds as C18:2 (n-6) levels were the same in both feeds. Our results suggest a potential mobilization of FA from the liver and not from muscle as an energy provider in fish-fed experimental yeast feed. The difference in FA composition observed in the liver tissue and not in the muscle tissue between the two fish groups may indicate a compensation of the liver from the experimental fish group to cover the lipid requirements in the muscle. A change of FA composition in the liver could lead to deleterious effects on the health of the fish which would bring to light differences in growth parameters, histology, and metabolic studies.

Both liver weight and HSI were significantly higher in fish fed with the experimental feed despite a non-statistical difference in liver fat content and liver n-3/n-6 ratio in the two groups of fish. Elevated HSI could indicate a disorder or injury in the liver related to an increase of glycogen or lipid storage which can be the result of the high content of lipids and carbohydrates in the feed or exposure to contaminants (Nayak et al., 1996). Related to our results, the analysis of several organic pollutants (polycyclic aromatic hydrocarbons, polychlorinated biphenyls, and hexachlorobenzene) and heavy metals (aluminum, arsenic, cadmium, mercury, and lead) in experimental feed was carried out and values were found below the European legislation (manuscript in preparation). The histological analysis of fish livers from both control and experimental groups revealed no significant difference in the number of lipid droplets. However, the phenotypical results have shown larger lipid droplets in the fish having the highest liver fat content. Some experimental fish have shown a higher fat content than control fish but the results were not consistent and not significant.

Regarding high carbohydrate content in fish feed, several studies have shown an increase in liver glycogen content, HSI value, or lipid droplets with increased dietary starch or carbohydrate levels (Wilson, 1994; Moreira et al., 2008; Prathomya et al., 2017; Zhao et al., 2022). Carnivorous fish such as salmonids are limited in their dietary carbohydrates from their environment and have a lower tolerance for feed rich in carbohydrates compared to omnivorous or herbivorous species (Hemre, Mommsen & Krogdahl, 2002; Moreira et al., 2008). A lower tolerance for a high carbohydrate diet can lead to changes in hepatic glycolysis and gluconeogenesis regulation with for example a significant increase of glycogen content in the liver of rainbow trout fed with a high carbohydrate diet in addition to higher gene expression levels of genes encoding for glucose transporters and glycolytic enzymes (Song et al., 2018). To investigate the different energy pathways of fish in a broad range, we applied a metabolomics method using ^1^H-NMR and quantified metabolites in the aqueous phase of fish livers from both groups.

Surprisingly, no metabolites including glucose and lactose were significantly different in the liver between the control and experimental fed fish. Differences were expected in levels of metabolites involved in carbohydrate and lipid storage metabolism to account for the elevated HSI and liver weight in fish fed with experimental yeast feed.

Metabolites involved in the citric acid cycle (TCA cycle) such as succinate and fumarate and precursors of the TCA cycle such as glycerol were slightly lower in the liver of fish fed with experimental feed while pyruvate levels were similar. These results could indicate that intermediates of the TCA cycle were not produced at similar rates in the experimental group of fish compared to the control group or mobilized from the liver to the blood flow for gluconeogenesis activity. A previous study by Geisler et al. (2016) showed an increase of hepatic phosphoenolpyruvate carboxykinase (enzyme activity and mRNA expression), another intermediate of the TCA cycle and gluconeogenic potential, with a fasting period of 8 hours in mice. In addition, serum levels of 3-hydroxybutyrate in mice were found to be elevated from 8 hours of fasting and continued to increase with longer fasting time (Geisler et al., 2016). Similar results appear in our study in fish-fed experimental feed as plasma levels of 3-hydroxybutyrate were significantly higher compared to the control group of fish.

The potential gluconeogenesis activity observed in the metabolomics analysis of the liver of experimental fed fish can be supported by the plasma metabolic profile with significantly higher plasma lactate, alanine, slightly higher but non-significant glucose and pyruvate contents, and a nearly significant increase of glutamine, all of which are important gluconeogenic substrates (deRoos et al., 1985). An early study (Mommsen, French & Hochachka, 1980) suggested that a primary function of alanine in Atlantic salmon was to act as an amino acid carrier into the blood to be metabolized in other tissues for gluconeogenesis. Kullgren et al. (2010) found elevated levels of alanine in the muscle of fasting rainbow trout and a decrease in the liver of the same fish. The authors suggested two different functions of alanine with the first role as a substrate for hepatic glycogen and glucose production and the second one as an energy provider through oxidation in the liver. In our study, alanine levels in the liver remained unchanged between the two fish groups with similar observations for glucose, glutamate, and lactate levels.

Metabolites involved in the one-carbon (1C) metabolism, choline, betaine, N-N, dimethylglycine, glycine, and serine were all significantly higher in plasma of fish fed with experimental feed, as was methionine, but in a non-significant way. The liver is the main site of lipid and one-carbon metabolisms (da Silva, Eudy & Deminice, 2020) and low concentrations of metabolites involved in the one-carbon metabolism have been associated with fat accumulation in the liver of humans and mice, as well as a progression of liver disease such as nonalcoholic fatty liver disease (da Silva, Eudy & Deminice, 2020). Minor alterations of the one-carbon metabolism observed in our study suggest that experimental fish should not show signs of liver damage or fat accumulation. In line with this, no damaged tissue was observed in the histological results of fish livers for both groups. Lipid content was slightly higher in experimental fish although fat accumulation in regards to the number and size of lipid droplets could not be seen consistently in a specific fish group.

Plasma creatinine levels were significantly higher in fish fed with experimental feed. A higher level of creatinine was observed in the plasma of gilthead sea bream fish fed once a day with fishmeal and fish oil compared to fish fed the same meal multiple times a day. The effect observed in plasma creatinine was attributed to a faster protein metabolism (Busti et al., 2020). Another factor potentially influencing plasma creatinine levels is the disruption of the kidney function in fish fed with experimental feed as kidneys are responsible for the excretion of creatinine (Stoskopf, 1993). Exposure to contaminants or pesticides can be a cause of interferences in fish kidney function (Taheri Mirghaed et al., 2018) which would be revealed with histology studies of vital organs and quantification of plasma urea levels.

Propylene glycol (PG, 1,2-propanediol) is a synthetic diol incorporated in animal feed as an antifoaming agent or to improve plasticity, and texture and can act as an antimicrobial or antifreeze agent (Hilton, Atkinson, and Slinger, 1986; Nalawade, Bhat & Sogi 2015; Soaudy et al., 2021). In our study, PG was detected in the plasma of fish fed with experimental feed as the yeast production included the addition of polypropylene glycol as an antifoaming agent. No detrimental effect of dietary PG on fish health has been observed in rainbow trout (Hilton, Atkinson & Slinger, 1986), or in Atlantic salmon (Aru et al., 2021). An increase in survival, growth performance, feed utilization, and improved immune response were identified in Nile tilapia after dietary inclusion of PG to mitigate winter stress (Soaudy et al., 2021).

In relation to fish feed and interferences with energy pathways, Prathomya et al. (2017) demonstrated that a high-fat-high-carbohydrate diet fed to *Megalobrama amblycephala* induced the accumulation of amino acids leucine, isoleucine, valine, glutamine, histidine, methionine, and tyrosine in plasma. High levels of these amino acids in plasma were explained by a “congestion” of the TCA cycle due to an excess of products from carbohydrate and lipid metabolisms, reducing the number of metabolites entering the TCA cycle. Another type of diet included in the same study, a high carbohydrate diet, showed distinct signs of liver injuries, higher plasma levels of tyrosine and creatine, and no TCA cycle disruption. Our study indicates a disruption of the TCA cycle as well with a significant accumulation of plasma alanine, glycine, and serine involved in the one-carbon metabolic pathway and slightly lower levels of fumarate and succinate in the liver.

In addition to the interferences seen in our study with the TCA cycle, elevated plasma ketone bodies and gluconeogenesis activity observed in the liver and plasma of the fish fed with experimental feed suggest an unmet energy requirement in the experimental group of fish possibly due to indigestible compounds from the yeast biomass, which would translate into elevated levels of plasma metabolites involved in a fasting state.

The implementation of metabolomics tools to understand fish physiology with novel feeds brings new knowledge to aquaculture. The quantification of metabolites related to feeding intake provides more appropriate information about fish nutrition and leads to possibilities for fish health improvement (Lulijwa, Alfaro & Young, 2022). The welfare of fish when modifying the feed to more sustainable sources should be of importance. Among common metabolomics tissue and fluid samples, plasma samples are suited in the study of fish nutrition as plasma analysis provides a snapshot of different metabolic activities occurring in a system, since plasma is involved in the transport of nutrients and diverse signaling molecules to organs. Nevertheless, the measurement of plasma metabolites is not isolated from effects related to nutrition and other biological factors such as stress, diseases, and growth can confuse results (Roques et al., 2020).

The composition of both feeds in this study was designed to contain similar proportions of macronutrients with the only difference being the inclusion of yeast biomass as a new ingredient. Nevertheless, information on the exact composition of the yeast cell walls from *R. toruloides* is scarce (Buck & Andrews, 1999; Yockey et al., 2019). One study (Weijman, de Miranda & Van Der Walt, 1988) differentiated *Rhodotorula* from *Cryptococcus* yeasts by having high amounts of mannan and no amount of xylose in the cell wall, and a lower urease activity.

The carbohydrate composition of *R. toruloides* has been previously described with a major quantity of mannose and glucose followed by smaller amounts of galactose and very low amounts of rhamnose, fucose, ribose, arabinose, and no amount of xylose (Sugiyama et al., 1985). Cell walls of *R. toruloides* in our study were physically disrupted while applying a French press before incorporating the yeast biomass into the experimental fish feed. Physical disruption was applied in order to facilitate the uptake of yeast nutrients by the fish. This might also have resulted in increased bioavailability of the carbohydrates from the cell walls.

Most yeasts contain diverse immune-stimulating compounds, such as β-glucans, mannan-oligosaccharides, and chitin (Navarrete & Tovar-Ramírez, 2014) whose incorporation in fish feed could potentially improve fish growth performance, immune system, feed efficiency, stress tolerance and intestinal microbiota (Navarrete & Tovar-Ramírez, 2014; Rimoldi et al., 2020; Zhang et al., 2020). The beneficial or detrimental effects of yeasts on fish health as an ingredient in fish feed seem to be linked to multiple factors including the type of yeasts, the quantity added to the feed, and the fish species (Øverland et al. ., 2013; Navarrete and Tovar-Ramírez, 2014; Øverland & Skrede, 2017; Blomqvist et al., 2018; Sahlmann et al., 2019; Vidakovic et al., 2020). More research should be conducted on the composition of different yeast species and their inclusion in fish feed for aquaculture purposes.

## 5 Conclusion

The replacement of vegetable oils with yeast biomass in Arctic char feed did not lead to differences in the fat content of muscle and liver tissues. The fatty acid profiles were similar in muscle tissue and showed minor variations in the liver tissue. Higher liver weight and HSI were observed in fish fed with experimental feed along with increased plasma ketones and metabolites involved in the one-carbon metabolism. No significant difference in the number of lipid droplets was seen and droplet sizes were consistent with fat content at the individual level. Further investigations are required before using *R. toruloides* extensively in fish feed.

## Data Availability

The original contributions presented in the study are included in the article/Supplementary Material, further inquiries can be directed to the corresponding author.

## References

[B1] Abeln F. Chuck C. J. (2021). The history, state of the art and future prospects for oleaginous yeast research. Microb. Cell Fact. 20, 221. 10.1186/s12934-021-01712-1 34876155 PMC8650507

[B2] Appelqvist L.-Å. (1968). Rapid methods of lipid extraction and fatty acid methyl ester preparation for seed and leaf tissue with special remarks on preventing the accumulation of lipid contaminants. Ark. för Kemi 28, 551–570.

[B3] Aru V. Khakimov B. Sørensen K. M. Chikwati E. M. Kortner T. M. Midtlyng P. (2021). The plasma metabolome of Atlantic salmon as studied by ^1^H NMR spectroscopy using standard operating procedures: Effect of aquaculture location and growth stage. Metabolomics. 17, 50. 10.1007/s11306-021-01797-0 33999285

[B4] Blomqvist J. Pickova J. Tilami S. K. Sampels S. Mikkelsen N. Brandenburg J. (2018). Oleaginous yeast as a component in fish feed. Sci. Rep. 8, 15945. 10.1038/s41598-018-34232-x 30374026 PMC6206134

[B5] Bradbury J. (2011). Docosahexaenoic acid (DHA): An ancient nutrient for the modern human brain. Nutrients 3, 529–554. 10.3390/nu3050529 22254110 PMC3257695

[B6] Brandenburg J. Blomqvist J. Shapaval V. Kohler A. Sampels S. Sandgren M. (2021). Oleaginous yeasts respond differently to carbon sources present in lignocellulose hydrolysate. Biotechnol. Biofuels 14, 124. 10.1186/s13068-021-01974-2 34051838 PMC8164748

[B7] Buck J. W. Andrews J. H. (1999). Attachment of the yeast *Rhodosporidium toruloides* is mediated by adhesives localized at sites of bud cell development. Appl. Environ. Microbiol. 65, 465–471. 10.1128/aem.65.2.465-471.1999 9925569 PMC91048

[B8] Busti S. Bonaldo A. Dondi F. Cavallini D. Yúfera M. Gilannejad N. (2020). Effects of different feeding frequencies on growth, feed utilisation, digestive enzyme activities and plasma biochemistry of gilthead sea bream (*Sparus aurata*) fed with different fishmeal and fish oil dietary levels. Aquaculture 529, 735616. 10.1016/j.aquaculture.2020.735616

[B9] Casu F. Watson A. M. Yost J. Leffler J. W. Gaylord T. G. Barrows F. T. (2017). Metabolomics analysis of effects of commercial soy-based protein products in red drum (*Sciaenops ocellatus*). J. Proteome Res. 16, 2481–2494. 10.1021/acs.jproteome.7b00074 28613908 PMC5604330

[B10] Cheng K. Müllner E. Moazzami A. A. Carlberg H. Brännäs E. Pickova J. (2017). Metabolomics approach to evaluate a Baltic Sea sourced diet for cultured Arctic char (*Salvelinus alpinus* L.). J. Agric. Food Chem. 65, 5083–5090. 10.1021/acs.jafc.7b00994 28557427

[B11] Cheng K. Wagner L. Moazzami A. A. Gómez-Requeni P. Schiller Vestergren A. Brännäs E. (2016a). Decontaminated fishmeal and fish oil from the Baltic Sea are promising feed sources for Arctic char (*Salvelinus alpinus* L.)-studies of flesh lipid quality and metabolic profile. Eur. J. Lipid Sci. Technol. 118, 862–873. 10.1002/ejlt.201500247

[B12] Cheng K. Wagner L. Pickova J. Moazzami A. A. (2016b). NMR-based metabolomics reveals compartmental metabolic heterogeneity in liver of Arctic char (*Salvelinus alpinus*). Can. J. Zool. 94, 665–669. 10.1139/cjz-2016-0051

[B13] da Silva R. P. Eudy B. J. Deminice R. (2020). One-carbon metabolism in fatty liver disease and fibrosis: One-carbon to rule them all. J. Nutr. 150, 994–1003. 10.1093/jn/nxaa032 32119738

[B14] de Roos B. Sneddon A. A. Sprague M. Horgan G. W. Brouwer I. A. (2017). The potential impact of compositional changes in farmed fish on its health-giving properties: Is it time to reconsider current dietary recommendations? Public Health Nutr. 20, 2042–2049. 10.1017/S1368980017000696 28535834 PMC10261345

[B15] deRoos R. deRoos C. C. Werner C. S. Werner H. (1985). Plasma levels of glucose, alanine, lactate, and β-hydroxybutyrate in the unfed spiny dogfish shark (*Squalus acanthias*) after surgery and following mammalian insulin infusion. Gen. Comp. Endocrinol. 58, 28–43. 10.1016/0016-6480(85)90133-9 3886476

[B16] Dupont-Cyr B. A. Le François N. R. Christen F. Desrosiers V. Savoie A. Vandenberg G. W. (2022). Linseed oil as a substitute for fish oil in the diet of Arctic charr (*Salvelinus alpinus*), brook charr (*S. fontinalis*) and their reciprocal hybrids. Aquac. Rep. 22, 100949. 10.1016/j.aqrep.2021.100949

[B17] Eriksson L. Byrne T. Johansson E. Trygg J. Vikström C. (2013). Multi- and megavariate data analysis, basic principles and applications. Malmö MKS Umetrics AB, 521.

[B18] Eriksson L. Trygg J. Wold S. (2008). CV-ANOVA for significance testing of PLS and OPLS® models. J. Chemom. 22, 594–600. 10.1002/cem.1187

[B19] FAO (2020). The state of world fisheries and aquaculture 2020: Sustainability in action. Rome. 10.4060/ca9229en

[B20] Fry J. P. Love D. C. MacDonald G. K. West P. C. Engstrom P. M. Nachman K. E. (2016). Environmental health impacts of feeding crops to farmed fish. Environ. Int. 91, 201–214. 10.1016/j.envint.2016.02.022 26970884

[B21] Gamboa‐Delgado J. Márquez-Reyes J. M. (2018). Potential of microbial‐derived nutrients for aquaculture development. Rev. Aquac. 10, 224–246. 10.1111/raq.12157

[B22] Geisler C. E. Hepler C. Higgins M. R. Renquist B. J. (2016). Hepatic adaptations to maintain metabolic homeostasis in response to fasting and refeeding in mice. Nutr. Metab. 13, 62. 10.1186/s12986-016-0122-x PMC5037643 27708682

[B23] Hara A. Radin N. S. (1978). Lipid extraction of tissues with a low-toxicity solvent. Anal. Biochem. 90, 420–426. 10.1016/0003-2697(78)90046-5 727482

[B24] Hatlen B. Berge M. G. Odom J. M. Mundheim H. Ruyter B. (2012). Growth performance, feed utilisation and fatty acid deposition in Atlantic salmon , *Salmo salar* L., fed graded levels of high-lipid/high-EPA *Yarrowia lipolytica* biomass. Aquaculture 364–365, 39–47. 10.1016/j.aquaculture.2012.07.005

[B25] Hemre G. I. Mommsen T. P. Krogdahl Å. (2002). Carbohydrates in fish nutrition: Effects on growth, glucose metabolism and hepatic enzymes. Aquac. Nutr. 8, 175–194. 10.1046/j.1365-2095.2002.00200.x

[B26] Hilton J. W. Atkinson J. L. Slinger S. J. (1986). Effect of propylene glycol on feed digestibility and the growth and physiological response of rainbow trout. Can. J. Anim. Sci. 66, 1057–1063. 10.4141/cjas86-116

[B27] Kalogeropoulos N. Alexis M. N. Henderson R. J. (1992). Effects of dietary soybean and cod-liver oil levels on growth and body composition of gilthead bream (*Sparus aurata*). Aquaculture 104, 293–308. 10.1016/0044-8486(92)90211-3

[B28] Kullgren A. Samuelsson L. M. Larsson D. G. J. Björnsson B. T. Bergman E. J. (2010). A metabolomics approach to elucidate effects of food deprivation in juvenile rainbow trout (*Oncorhynchus mykiss*). Am. J. Physiol. Regul. Integr. Comp. Physiol. 299, 1440–1448. 10.1152/ajpregu.00281.2010 20861281

[B29] Lulijwa R. Alfaro A. C. Young T. (2022). Metabolomics in salmonid aquaculture research: Applications and future perspectives. Rev. Aquac. 14, 547–577. 10.1111/raq.12612

[B30] Moazzami A. A. Andersson R. Kamal-Eldin A. (2011). Changes in the metabolic profile of rat liver after α-tocopherol deficiency as revealed by metabolomics analysis. NMR Biomed. 24, 499–505. 10.1002/nbm.1615 21674651

[B31] Mommsen T. P. French C. J. Hochachka P. W. (1980). Sites and patterns of protein and amino acid utilization during the spawning migration of salmon. Can. J. Zool. 58, 1785–1799. 10.1139/z80-246

[B32] Monge-Ortiz R. Tomás-Vidal A. Rodriguez-Barreto D. Martínez-Llorens S. Pérez J. A. Jover-Cerdá M. (2018). Replacement of fish oil with vegetable oil blends in feeds for greater amberjack (*Seriola dumerili*) juveniles: Effect on growth performance, feed efficiency, tissue fatty acid composition and flesh nutritional value. Aquac. Nutr. 24, 605–615. 10.1111/anu.12595

[B33] Morales-Sánchez D. Martinez-Rodriguez O. A. Martinez A. (2017). Heterotrophic cultivation of microalgae: Production of metabolites of commercial interest. J. Chem. Technol. Biotechnol. 92, 925–936. 10.1002/jctb.5115

[B34] Moreira I. S. Peres H. Couto A. Enes P. Oliva-Teles A. (2008). Temperature and dietary carbohydrate level effects on performance and metabolic utilisation of diets in European sea bass (*Dicentrarchus labrax*) juveniles. Aquaculture 274, 153–160. 10.1016/j.aquaculture.2007.11.016

[B35] Mráz J. Pickova J. (2009). Differences between lipid content and composition of different parts of fillets from crossbred farmed carp (*Cyprinus carpio*). Fish. Physiol. Biochem. 35, 615–623. 10.1007/s10695-008-9291-5 19043793

[B36] Nagaraj Y. N. Burkina V. Okmane L. Blomqvist J. Rapoport A. Sandgren M. (2022). Identification, quantification and kinetic study of carotenoids and lipids in *Rhodotorula toruloides* CBS 14 cultivated on wheat straw hydrolysate. Fermentation 8, 300. 10.3390/fermentation8070300

[B37] Nalawade T. M. Bhat K. Sogi S. H. P. (2015). Bactericidal activity of propylene glycol, glycerine, polyethylene glycol 400, and polyethylene glycol 1000 against selected microorganisms. J. Int. Soc. Prev. Community Dent. 5, 114–119. 10.4103/2231-0762.155736 25992336 PMC4415329

[B38] Navarrete P. Tovar-Ramírez D. (2014). Use of yeasts as probiotics in fish aquaculture. Sustain. Aquac. Tech. 10.5772/57196

[B39] Nayak N. C. Sathar S. A. Mughal S. Duttagupta S. Mathur M. Chopra P. (1996). The nature and significance of liver cell vacuolation following hepatocellular injury - an analysis based on observations on rats rendered tolerant to hepatotoxic damage. Virchows Arch. 428, 353–365. 10.1007/BF00202202 8797939

[B40] Øverland M. Karlsson A. Mydland L. T. Romarheim O. H. Skrede A. (2013). Evaluation of *Candida utilis*, *Kluyveromyces marxianus* and *Saccharomyces cerevisiae* yeasts as protein sources in diets for Atlantic salmon (*Salmo salar*). Aquaculture 402-403, 1–7. 10.1016/j.aquaculture.2013.03.016

[B41] Øverland M. Skrede A. (2017). Yeast derived from lignocellulosic biomass as a sustainable feed resource for use in aquaculture. J. Sci. Food Agric. 97, 733–742. 10.1002/jsfa.8007 27558451

[B42] Pettersson A. Johnsson L. Brännäs E. Pickova J. (2009). Effects of rapeseed oil replacement in fish feed on lipid composition and self-selection by rainbow trout (*Oncorhynchus mykiss*). Aquac. Nutr. 15, 577–586. 10.1111/j.1365-2095.2008.00625.x

[B43] Pettersson A. Pickova J. Brännäs E. (2010). Swimming performance at different temperatures and fatty acid composition of Arctic charr (*Salvelinus alpinus*) fed palm and rapeseed oils. Aquaculture 300, 176–181. 10.1016/j.aquaculture.2010.01.017

[B44] Pickova J. Dutta P. C. Larsson P.-O. Kiessling A. (1997). Early embryonic cleavage pattern, hatching success, and egg-lipid fatty acid composition: Comparison between two cod (*Gadus morhua*) stocks. Can. J. Fish. Aquat. Sci. 54, 2410–2416. 10.1139/f97-148

[B45] Pinheiro M. J. Bonturi N. Belouah I. Miranda E. A. Lahtvee P. J. (2020). Xylose metabolism and the effect of oxidative stress on lipid and carotenoid production in *Rhodotorula toruloides*: Insights for future biorefinery. Front. Bioeng. Biotechnol. 8, 1008. 10.3389/fbioe.2020.01008 32974324 PMC7466555

[B46] Prathomya P. Prisingkorn W. Jakovlić I. Deng F. Y. Zhao Y. H. Wang W. M. (2017). ^1^H NMR-based metabolomics approach reveals metabolic alterations in response to dietary imbalances in *Megalobrama amblycephala* . Metabolomics 13, 17. 10.1007/s11306-016-1158-7

[B47] Rimoldi S. Gini E. Koch J. F. A. Iannini F. Brambilla F. Terova G. (2020). Correction to: Effects of hydrolyzed fish protein and autolyzed yeast as substitutes of fishmeal in the gilthead sea bream (*Sparus aurata*) diet, on fish intestinal microbiome. BMC Vet. Res. 16, 219. 10.1186/s12917-020-02416-1 32600314 PMC7322861

[B48] Röhnisch H. E. Eriksson J. Müllner E. Agback P. Sandström C. Moazzami A. A. (2018). AQuA: An automated quantification algorithm for high-throughput NMR-based metabolomics and its application in human plasma. Anal. Chem. 90, 2095–2102. 10.1021/acs.analchem.7b04324 29260864

[B49] Roques S. Deborde C. Richard N. Skiba-Cassy S. Moing A. Fauconneau B. (2020). Metabolomics and fish nutrition: A review in the context of sustainable feed development. Rev. Aquac. 12, 261–282. 10.1111/raq.12316

[B50] Sahlmann C. Djordjevic B. Lagos L. Mydland L. T. Morales-Lange B. Hansen J. Ø. (2019). Yeast as a protein source during smoltification of Atlantic salmon (*Salmo salar* L.), enhances performance and modulates health. Aquaculture 513, 734396. 10.1016/j.aquaculture.2019.734396

[B51] Sánchez-Vázquez F. J. Yamamoto T. Akiyama T. Madrid J. A. Tabata M. (1999). Macronutrient self-selection through demand-feeders in rainbow trout. Physiol. Behav. 66, 45–51. 10.1016/S0031-9384(98)00313-8 10222472

[B52] Shah M. R. Lutzu G. A. Alam A. Sarker P. Kabir Chowdhury M. A. Parsaeimehr A. (2018). Microalgae in aquafeeds for a sustainable aquaculture industry. J. Appl. Phycol. 30, 197–213. 10.1007/s10811-017-1234-z

[B53] Soaudy M. R. Mohammady E. Y. Elashry M. A. Ali M. M. Ahmed N. M. Hegab M. H. (2021). Possibility mitigation of cold stress in Nile tilapia under biofloc system by dietary propylene glycol: Performance feeding status, immune, physiological responses and transcriptional response of delta-9-desaturase gene. Aquaculture 538, 736519. 10.1016/j.aquaculture.2021.736519

[B54] Song X. Marandel L. Skiba-Cassy S. Corraze G. Dupont-Nivet M. Quillet E. (2018). Regulation by dietary carbohydrates of intermediary metabolism in liver and muscle of two isogenic lines of rainbow trout. Front. Physiol. 9, 1579. 10.3389/fphys.2018.01579 30483148 PMC6243097

[B55] Sprague M. Betancor M. B. Tocher D. R. (2017). Microbial and genetically engineered oils as replacements for fish oil in aquaculture feeds. Biotechnol. Lett. 39, 1599–1609. 10.1007/s10529-017-2402-6 28721583 PMC5636849

[B56] Stoskopf M. K. (1993). Fish medicine. Saint Louis, MO: WB Saunders Company.

[B57] Sugiyama J. Fukagawa M. Chiu S. W. Komagata K. (1985). Cellular carbohydrate composition, DNA base composition, ubiquinone systems, and diazonium blue B color test in the genera *Rhodosporidium, Leucosporidium, Rhodotorula* and related basidiomycetous yeasts. J. Gen. Appl. Microbiol. 31, 519–550. 10.2323/jgam.31.519

[B58] Taheri Mirghaed A. Ghelichpour M. Mirzargar S. S. Joshaghani H. Ebrahimzadeh Mousavi H. (2018). Toxic effects of indoxacarb on gill and kidney histopathology and biochemical indicators in common carp (*Cyprinus carpio*). Aquac. Res. 49, 1616–1627. 10.1111/are.13617

[B59] Teoh C. Y. Ng W. K. (2016). The implications of substituting dietary fish oil with vegetable oils on the growth performance, fillet fatty acid profile and modulation of the fatty acid elongase, desaturase and oxidation activities of red hybrid tilapia, *Oreochromis* sp. Aquaculture 465, 311–322. 10.1016/j.aquaculture.2016.09.023

[B60] Thomassen M. S. Røsjø C. (1989). Different fats in feed for salmon: Influence on sensory parameters, growth rate and fatty acids in muscle and heart. Aquaculture 79, 129–135. 10.1016/0044-8486(89)90453-5

[B61] Vidakovic A. Huyben D. Sundh H. Nyman A. Vielma J. Passoth V. (2020). Growth performance, nutrient digestibility and intestinal morphology of rainbow trout (*Oncorhynchus mykiss*) fed graded levels of the yeasts *Saccharomyces cerevisiae* and *Wickerhamomyces anomalus* . Aquac. Nutr. 26, 275–286. 10.1111/anu.12988

[B62] Wagner L. Gómez-Requeni P. Moazzami A. A. Lundh T. Vidakovic A. Langeland M. (2019). ^1^H NMR-based metabolomics and lipid analyses revealed the effect of dietary replacement of microbial extracts or mussel meal with fish meal to Arctic charr (*Salvelinus alpinus*). Fishes 4, 46. 10.3390/fishes4030046

[B63] Wagner L. Trattner S. Pickova J. Gómez-Requeni P. Moazzami A. A. (2014). ¹H NMR-based metabolomics studies on the effect of sesamin in Atlantic salmon (*Salmo salar*). Food Chem. 147, 98–105. 10.1016/j.foodchem.2013.09.128 24206691

[B64] Weijman A. C. M. Rodrigues de Miranda L. Van Der Walt J. P. (1988). Redefinition of *Candida* Berkhout and the consequent emendation of *Cryptococcus* Kützing and *Rhodotorula* Harrison. Antonie van Leeuwenhoek 54, 545–553. 10.1007/BF00588390 3232972

[B65] Willora F. P. Grønevik B. Liu C. Palihawadana A. Sørensen M. Hagen Ø. (2021). Total replacement of marine oil by rapeseed oil in plant protein rich diets of juvenile lumpfish (*Cyclopterus lumpus*): Effects on growth performance, chemical and fatty acid composition. Aquac. Rep. 19, 100560. 10.1016/j.aqrep.2020.100560

[B66] Wilson R. P. (1994). Utilization of dietary carbohydrate by fish. Aquaculture 124, 67–80. 10.1016/0044-8486(94)90363-8

[B67] Yockey J. Andres L. Carson M. Ory J. J. Reese A. J. (2019). Cell envelope integrity and capsule characterization of *Rhodotorula mucilaginosa* strains from clinical and environmental sources. MSphere 4, e00166–19. 10.1128/msphere.00166-19 31167944 PMC6553552

[B68] Zhang P. Yang F. Hu J. Han D. Liu H. Jin J. (2020). Optimal form of yeast cell wall promotes growth, immunity and disease resistance in gibel carp (*Carassius auratus* gibelio). Aquac. Rep. 18, 100465. 10.1016/j.aqrep.2020.100465

[B69] Zhao L. Liao L. Tang X. Liang J. Liu Q. Luo W. (2022). High-carbohydrate diet altered conversion of metabolites, and deteriorated health in juvenile largemouth bass. Aquaculture 549, 737816. 10.1016/j.aquaculture.2021.737816

