# Corrigendum: Oleaginous yeast *Rhodotorula toruloides* biomass effect on the metabolism of Arctic char (*Salvelinus alpinus*)

**DOI:** 10.3389/fmolb.2022.1101980

**Published:** 2022-12-12

**Authors:** Mathilde Brunel, Viktoriia Burkina, Jana Pickova, Sabine Sampels, Ali A. Moazzami

**Affiliations:** ^1^ Department of Molecular Sciences, Faculty of Natural Resources and Agricultural Sciences, Swedish University of Agricultural Sciences, Uppsala, Sweden; ^2^ Faculty of Fisheries and Protection of Waters, South Bohemian Research Center of Aquaculture and Biodiversity of Hydrocenoses, Research Institute of Fish Culture and Hydrobiology, University of South Bohemia in České Budějovice, Vodňany, Czechia

**Keywords:** metabolomics, fish feed replacement, fatty acids, metabolites, gluconeogenesis, plasma, liver, oleaginous yeast

In the published article, there was an error in Table 1 as published. The corrected Table 1 and its caption “Table 1 Composition of control and experimental yeast feeds (g kg^−1^) for fish in duplicates. “Vitamin mix” and “Mineral mix” ingredients were provided by NOFIMA (Norway) and “astaxanthin and vitamin mix” ingredients were provided by Aller Aqua A/S (Denmark)” appear below.

**TABLE 1:** Composition of control and experimental yeast feeds (g kg^−1^) for fish in duplicates. “Vitamin mix” and “Mineral mix” ingredients were provided by NOFIMA (Norway) and “astaxanthin and vitamin mix” ingredients were provided by Aller Aqua A/S (Denmark) appear below.

Ingredients	Control feed	Experimental feed
Fish meal	4,950	4,950
Fish oil	1,170	1,170
Vegetable oil	540	–
Mineral mix	45	45
Vitamin mix	90	90
Astaxanthin and vitamin mix	13.5	13.5
Gelatine	45	45
Wheat meal	1.755	1.305
Casein	540	–
Ca_2_PO_4_	225	225
Yeast	-	1,413 (540 g oil)

In the published article, there was an error in the legend for Table 4 as published. The corrected Table 4 and its caption “Table 4 Growth parameters for fish fed with control feed or experimental yeast-based feed. Data are presented as the mean ± standard deviation. Statistical significance was set at *p*-value < 0.05” appear below.

**TABLE 4:** Growth parameters for fish fed with control feed or experimental yeast-based feed. Data are presented as the mean ± standard deviation. Statistical significance was set at *p*-value < 0.05.

Growth parameters	Control group	Experimental group	*p*-value
Weight (g), day 19	209 ± 65.4	200 ± 62.9	0.7360
(n = 12 per group)			
Final length (cm)	29.4 ± 2.20	29.2 ± 2.54	0.7417
(n = 48 per group)			
Final weight (g)	314 ± 99.1	303 ± 94.3	0.6108
(n = 48 per group)			
**Liver weight (g)**	**3.75 ± 2.07**	**4.61 ± 1.91**	**0.0069**
(n = 48 per group)			
SGR (%)	0.68 ± 0.59	0.70 ± 0.56	0.8455
(53 days)			
CF (%)	1.20 ± 0.14	1.19 ± 0.22	0.9078
(n = 48 per group)			
**HSI (%)**	**1.14 ± 0.27**	**1.51 ± 0.34**	**<0.0001**
(n = 48 per group)			

Significant results are shown in bold letters. SGR, specific growth rate; CF, condition factor; HSI, hepatosomatic index.

A correction has been made to **2 Materials and methods,** *2.2 Feeding trial and sample collection*.

“Arctic char (*n* = 126, both genders) over 2 years old were randomly assigned to six 1 m × 1 m water tanks (triplicate tanks per feed with 21 fish per tank) with access to a flow-through system of freshwater (10 L min^−1^ with a water depth of 20 cm) from Lake Ansjön at Kälarne Aquaculture Center North, Sweden.”

The corrected sentence appears below:

“Arctic char (*n* = 126, both genders, juveniles) were randomly assigned to six 1 m × 1 m water tanks (triplicate tanks per feed with 21 fish per tank) with access to a flow-through system of freshwater (10 L min^−1^ with a water depth of 20 cm) from Lake Ansjön at Kälarne Aquaculture Center North, Sweden.”

A correction has been made to **2 Materials and methods,** *2.2 Feeding trial and sample collection.*

“The specific growth rate (SGR) was calculated from day 19 of the trial due to technical problems occurring at the rearing station during the start of the trial.”

A correction has been made to **2.6 Statistics**.

“Multivariate data analysis was performed on liver and plasma metabolites using the SIMCA software (version 17; Umetrics, Umeå, Sweden).”

The corrected sentence appears below:

“Multivariate data analysis was performed on liver and plasma metabolites using the SIMCA software (version 17; Umetrics, Suite of Data Analytics Solutions, Sartorius).”

The authors apologize for this error and state that this does not change the scientific conclusions of the article in any way. The original article has been updated.

