# Supplementary material for: Oleaginous yeast Rhodotorula toruloides biomass effect on the metabolism of Arctic char (Salvelinus alpinus)
Source: Front Mol Biosci. 2022 Aug 16;9:931946. doi: 10.3389/fmolb.2022.931946 (PMC9425082; doi:10.3389/fmolb.2022.931946)
Supplement: Supplementary file 1 [file DataSheet1.pdf]

C

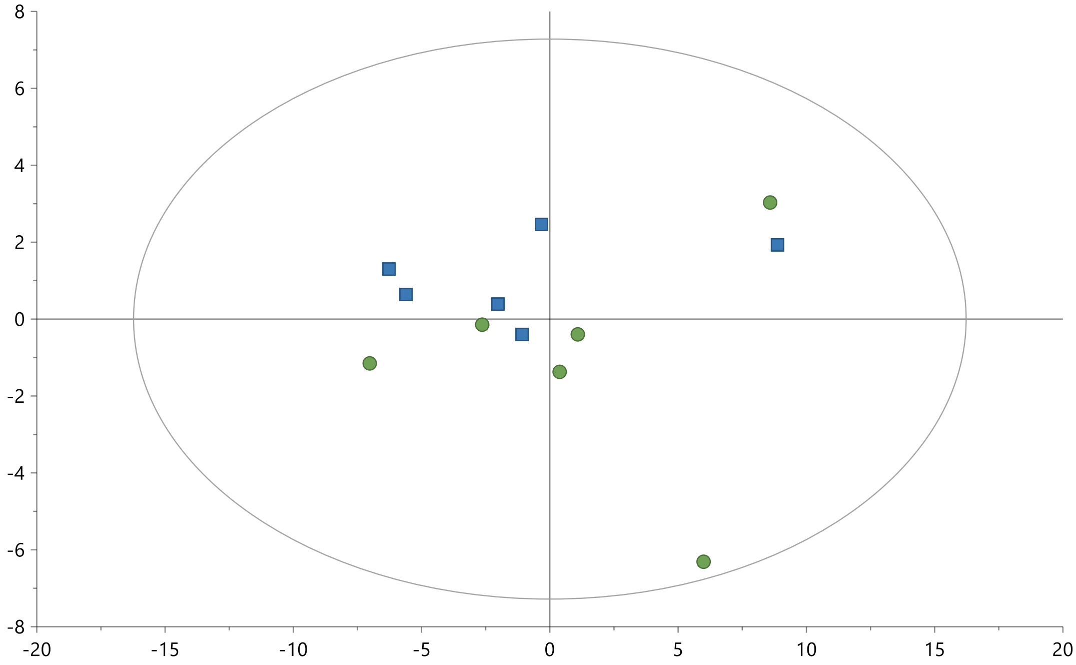

D

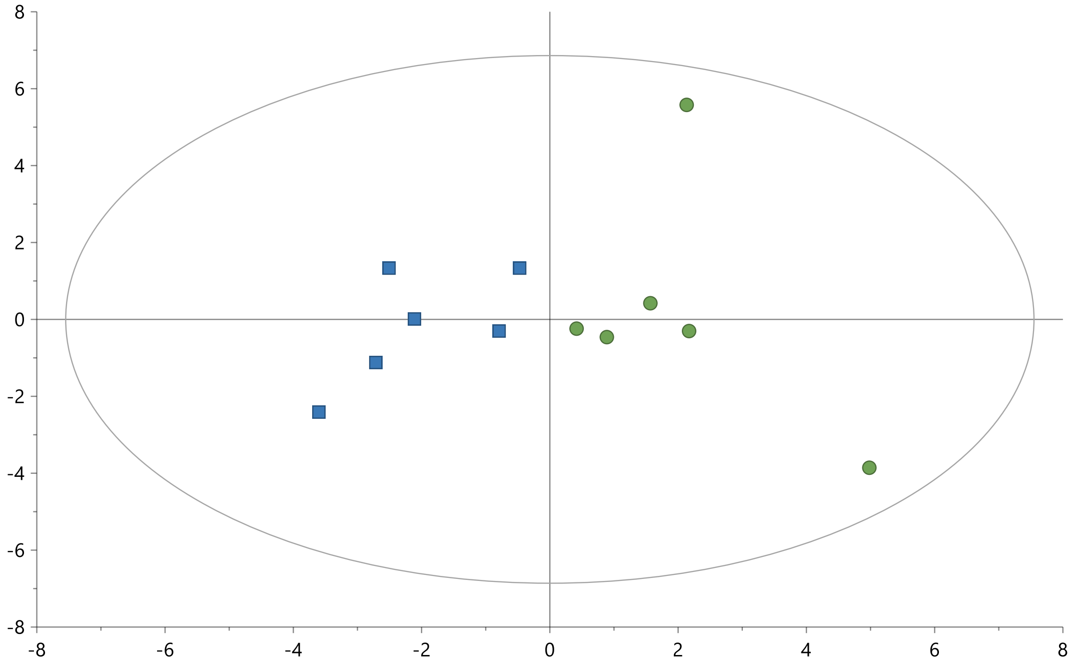

Supplementary Figure S1. Score plots of principal component analysis (PCA) and orthogonal partial least squares-discriminant analysis (OPLS-DA) with 1 predictive (P1) and 2 orthogonal (O2) components) models of ^{1}H-NMR aqueous liver tissue (n=12) and 48 metabolites. Green circles: fish fed control feed. Blue squares: fish fed with yeast biomass based feed. **C**- liver PCA-X score plot (all data, with 2 PCs) with parameters: R^2X =58.3 % , R^2X2 = 11.7 %, Q^2X = 44.3 %. **D**- liver OPLS-DA score plot (all data) with R^2X = 12.6 % Q^2X = 24.7 % R^2Y = 100% CV-ANOVA= 0.84.

Supplementary Table S1. Metabolites in liver (aqueous tissue, n=12), µM.g^{-1} (48 metabolites). Data are presented as median (Q1-Q3) with concentrations and p-value obtained from PROC MIXED function in SAS 9.4 with tank factor. Statistical significance was set at p-value <0.05.

	Concentration µM.g^{-1}		
Metabolites	Control fish (n=6)	Yeast fish (n=6)	p-value
3-Aminoisobutyrate	0.701 (0.438-0.771)	0.833 (0.144-1.209)	0.4542
ADP	0.014 (0.012-0.018)	0.016 (0.016-0.021)	0.7300
AMP	0.014 (0.012-0.015)	0.014 (0.012-0.016)	0.6510
ATP	0.031 (0.027-0.040)	0.035 (0.029-0.036)	0.8660
Acetate	0.124 (0.100-0.170)	0.150 (0.083-0.162)	0.9357
Acetone	0.073 (0.049-0.090)	0.066 (0.049-0.081)	0.6465
Alanine	4.241 (2.825-5.267)	4.220 (2.952-4.362)	0.9444
Asparagine	0.384 (0.240-0.496)	0.255 (0.226-0.352)	0.3378
Aspartate	1.149 (0.525-1.231)	0.435 (0.284-0.860)	0.1653
Betaine	0.073 (0.047-0.078)	0.088 (0.051-0.224)	0.7936
Cholate	0.157 (0.028-0.365)	0.055 (0.032-0.170)	0.3531
Choline	0.727 (0.491-0.808)	0.458 (0.355-0.735)	0.3894
Creatine	0.094 (0.051-0.109)	0.083 (0.049-0.124)	0.9549
Formate	0.291 (0.261-0.302)	0.323 (0.265-0.353)	0.2167
Fumarate	0.147 (0.080-0.201)	0.067 (0.053-0.086)	0.2361
Glucose	14.883 (11.589-20.842)	13.215 (9.783-19.119)	0.6278
Glutamate	2.674 (1.757-4.787)	2.112 (1.269-2.393)	0.4382
Glutamine	1.626 (1.153-2.351)	1.383 (0.891-1.592)	0.4426
Glutathione	0.146 (0.052-0.220)	0.128 (0.073-0.190)	0.8247
Glycerol	1.337 (0.966-1.553)	0.744 (0.612-0.993)	0.2581

Glycine	2.254 (1.604-3.063)	1.918 (1.205-2.013)	0.4917
Histamine	0.708 (0.340-0.813)	0.544 (0.287-0.571)	0.3969
Inosine	0.115 (0.079-0.161)	0.057 (0.047-0.179)	0.5474
Isoleucine	0.594 (0.391-0.642)	0.444 (0.228-0.516)	0.4342
Lactate	4.156 (2.821-4.972)	3.177 (2.730-5.374)	0.9284
Leucine	1.280 (0.843-1.564)	0.938 (0.530-1.049)	0.2700
Lysine	0.875 (0.642-1.024)	0.672 (0.459-1.025)	0.4911
Methanol	13.983 (11.819-15.063)	14.637 (3.414-27.075)	0.5650
Methionine	0.567 (0.349-0.602)	0.390 (0.207-0.450)	0.3263
N,N-Dimethylglycine	n.d	n.d	n.d
N-Methylhydantoin	n.d	n.d	n.d
NAD+	0.062 (0.058-0.076)	0.078 (0.066-0.088)	0.2470
NADP+	0.044 (0.043-0.053)	0.058 (0.050-0.072)	0.1218
Niacinamide	0.221 (0.203-0.303)	0.193 (0.165-0.290)	0.7628
O-Phosphocholine	0.145 (0.121-0.154)	0.120 (0.086-0.160)	0.5467
Phenylalanine	0.315 (0.235-0.403)	0.209 (0.162-0.310)	0.1703
Propionate	0.025 (0.012-0.030)	0.013 (0.003-0.015)	0.4914
Pyruvate	0.012 (0.010-0.014)	0.011 (0.010-0.012)	0.6817
S-Adenosylhomocysteine	0.043 (0.035-0.051)	0.031 (0.026-0.032)	0.2609
Sarcosine	0.016 (0.012-0.026)	0.018 (0.016-0.020)	0.5034
Succinate	0.184 (0.182-0.193)	0.087 (0.079-0.119)	0.3036
Taurine	12.664 (8.924-14.799)	9.662 (8.203-11.340)	0.4763
Tryptophan	0.288 (0.222-0.505)	0.151 (0.096-0.179)	0.0669
Tyrosine	0.426 (0.305-0.631)	0.256 (0.160-0.273)	0.2288
UDP-glucose	0.083 (0.058-0.158)	0.095 (0.074-0.117)	0.8955
UDP-glucuronate	0.064 (0.053-0.092)	0.084 (0.062-0.141)	0.3152
Uridine	0.024 (0.020-0.027)	0.021 (0.015-0.023)	0.5508
Valine	1.248 (0.787-1.316)	0.875 (0.470-1.051)	0.3365
sn-Glycero-3-phosphocholine	0.226 (0.167-0.349)	0.228 (0.151-0.389)	0.8120

ß-Alanine	0.414 (0.324-0.516)	0.418 (0.326-0.497)	0.9353

Supplementary Table S2. Metabolites in plasma (n=32 in total and 2 outliers in the treatment group), µM.L^{-1} (57 metabolites). Data are presented as median (Q1-Q3) with concentrations and p-value obtained from PROC MIXED function in SAS 9.4 with tank factor. Statistical significance was set at p-value <0.05. Results are presented without outliers fish 1 and fish 6.

	Concentrations µM.L^{-1}		
Metabolites	Control fish (n=16)	Yeast fish (n=14)	p-value
2-Aminobutyrate	33.36 (24.65-36.34)	29.96 (21.25-36.98)	0.5080
2-Hydroxybutyrate	15.51 (13.60-16.79)	16.58 (13.60-19.13)	0.2482
2-Hydroxyisovalerate	3.83 (3.19-4.25)	3.83 (2.55-4.25)	0.8802
2-Hydroxyvalerate	4.04 (3.19-4.89)	3.61 (2.98-5.10)	0.8454
2-Oxoisocaproate	2.13 (1.70-2.55)	2.34 (1.70-2.98)	0.4447
3-Hydroxybutyrate	5.53 (3.83-6.38)	11.69 (9.78-14.88)	0.0004
3-Methyl-2-oxovalerate	8.93 (7.65-10.20)	8.71 (7.65-11.05)	0.6842
Acetate	13.81 (12.54-16.36)	14.88 (12.33-17.43)	0.9478
Acetoacetate	16.79 (15.09-27.84)	15.51 (13.60-19.98)	0.1824
Acetone	3.40 (2.98-3.61)	3.40 (2.98-3.83)	0.7737
Alanine	480.25 (456.03-551.44)	758.41 (619.23-898.45)	0.0002
Arginine	214.84 (186.79-234.18)	226.31 (213.35-256.28)	0.1393
Asparagine	195.29 (169.15-208.46)	142.16 (110.93-205.70)	0.1693
Aspartate	24.23 (20.61-26.56)	24.23 (17.43-28.90)	0.7898
Betaine	30.60 (19.98-47.60)	47.39 (41.65-66.30)	0.0138
Carnitine	20.83 (18.06-34.00)	29.33 (23.38-35.28)	0.7866
Choline	46.11 (35.70-50.79)	58.23 (51.85-62.90)	0.0069
Citrate	71.61 (59.71-92.23)	65.45 (60.35-79.48)	0.4151
Creatine	326.61 (259.89-472.18)	444.98 (378.25-480.68)	0.3088
Creatinine	18.06 (15.94-21.25)	24.23 (22.53-25.50)	0.0005
Dimethyl sulfone	4.25 (3.83-4.68)	4.46 (4.25-5.10)	0.4066
Ethanol	22.31 (21.46-28.69)	28.26 (27.20-39.10)	0.2297
Formate	110.08 (106.04-114.96)	109.86 (107.95-117.73)	0.9845

Glucose	6558.39 (5947.88-7413.70)	6647.00 (6118.30-8195.28)	0.6418
Glutamate	113.05 (89.46-147.90)	105.19 (87.55-118.15)	0.3792
Glutamine	387.18 (349.78-422.66)	419.90 (362.53-450.08)	0.0631
Glycerol	351.05 (289.21-471.75)	362.95 (340.00-428.40)	0.6200
Glycine	440.51 (369.54-511.70)	544.64 (489.18-643.45)	0.0354
Hippurate	n.d.	n.d.	n.d.
Histidine	75.44 (60.14-87.13)	56.53 (51.00-65.45)	0.0765
Inosine	101.58 (82.24-128.14)	112.20 (103.28-233.33)	0.1157
Isoleucine	199.75 (177.65-214.63)	221.21 (162.78-243.95)	0.5071
Isopropanol	2.76 (2.13-42.71)	45.69 (3.40-55.68)	0.0757
Lactate	5371.15 (4376.01-6001.43)	6527.79 (5001.40-7247.10)	0.0413
Leucine	269.66 (247.56-283.05)	290.28 (227.80-314.08)	0.5939
Lysine	527.64 (443.49-574.60)	448.59 (359.13-521.05)	0.2223
Methanol	264.14 (245.44-294.74)	271.58 (249.90-285.60)	0.9372
Methionine	105.19 (84.58-116.88)	120.49 (100.30-150.88)	0.0157
N,N-Dimethylglycine	1.70 (1.70-2.13)	2.55 (1.70-3.40)	0.0413
N-Acetylcysteine	5.31 (3.19-8.71)	8.08 (5.53-11.05)	0.7394
O-Acetylcarnitine	4.46 (3.61-5.53)	5.95 (5.53-7.65)	0.0719
O-Phosphocholine	8.08 (5.95-9.35)	8.08 (7.23-10.20)	0.2573
Phenylalanine	97.96 (89.04-109.86)	91.38 (87.98-99.88)	0.1202
Proline	146.20 (131.54-157.89)	180.20 (154.28-192.53)	0.2482
Propionate	1.70 (1.28-2.13)	1.91 (0.85-2.98)	0.1809
Propylene glycol	6.38 (5.10-7.44)	29.54 (25.93-32.30)	<.0001
Pyruvate	12.96 (10.20-17.64)	17.64 (7.65-28.05)	0.4004
Sarcosine	1.28 (1.28-2.13)	1.70 (1.28-2.13)	0.2821
Serine	167.88 (151.09-198.90)	226.53 (185.30-240.98)	0.0132
Succinate	11.48 (9.35-17.43)	12.75 (9.35-17.85)	0.8491
Taurine	1420.56 (1234.63-1814.54)	1243.76 (1115.20-1341.30)	0.0630

Threonine	610.73 (426.28- 690.20)	461.76 (382.08-548.25)	0.1508
Trigonelline	n.d.	n.d.	n.d.
Trimethylamine N-oxide	400.35 (303.24- 497.89)	395.46 (314.08-535.93)	0.5229
Tyrosine	93.93 (77.56-107.95)	76.93 (64.60-80.33)	0.0089
Valine	459.21 (419.69-480.68)	453.26 (341.28-517.23)	0.5617
myo-Inositol	139.83 (132.39-168.94)	146.20 (127.50-153.85)	0.3391
trans-4-Hydroxy-L-proline	202.30 (164.26-239.49)	219.94 (177.65-260.10)	0.6920
β-Alanine	123.46 (100.30-149.60)	147.90 (124.53-162.78)	0.4989

Supplementary Table S3. Fat content (%) and number of lipid droplets in fish livers from control and experimental (yeast) groups (n= 8 in total, with 4 in each group). Lipid droplets were counted manually in ImageJ at the same area of (1.44 x 1.44) cm^2 using grid (line) option. Data are presented in the table by decreasing fat content (%).

**Tank**	**Feed group**	**Fish ID**	**Fat content (%)**	**Droplets number**
**179**	yeast	15	23.81	3.83
**181**	yeast	26	23.34	4.29
**178**	control	7	16.85	4.58
**182**	control	33	15.82	4.88
**177**	yeast	2	14.48	5.80
**181**	yeast	30	13.26	4.83
**182**	control	34	11.70	3.92
**178**	control	11	10.82	4.42
